# Molecular evolution and diversification of the GRF transcription factor family

**DOI:** 10.1590/1678-4685-GMB-2020-0080

**Published:** 2020-07-24

**Authors:** Leila Spagnolo Fonini, Fernanda Lazzarotto, Pedro M. Barros, Caroline Cabreira-Cagliari, Marcelo Affonso Begossi Martins, Nelson J.M. Saibo, Andreia Carina Turchetto-Zolet, Marcia Margis-Pinheiro

**Affiliations:** 1 Universidade Federal do Rio Grande do Sul, Centro de Biotecnologia, Programa de Pós-graduação em Biologia Celular e Molecular, Porto Alegre, RS, Brazil.; 2 Universidade Nova de Lisboa, Instituto de Tecnologia Química e Biológica António Xavier (ITQB NOVA), Oeiras, Portugal.; 3 Universidade Federal do Rio Grande do Sul, Departamento de Genética, Programa de Pós-Graduação em Genética e Biologia Molecular, Porto Alegre, RS, Brazil.

**Keywords:** GRF, molecular evolution, QLQ domain, Bayesian analysis

## Abstract

Abstract - Growth Regulating Factors (GRFs) comprise a transcription factor family with important functions in plant growth and development. They are characterized by the presence of QLQ and WRC domains, responsible for interaction with proteins and DNA, respectively. The QLQ domain is named due to the similarity to a protein interaction domain found in the SWI2/SNF2 chromatin remodeling complex. Despite the occurrence of the QLQ domain in both families, the divergence between them had not been further explored. Here, we show evidence for GRF origin and determined its diversification in angiosperm species. Phylogenetic analysis revealed 11 well-supported groups of GRFs in flowering plants. These groups were supported by gene structure, synteny, and protein domain composition. Synteny and phylogenetic analyses allowed us to propose different sets of probable orthologs in the groups. Besides, our results, together with functional data previously published, allowed us to suggest candidate genes for engineering agronomic traits. In addition, we propose that the QLQ domain of *GRF* genes evolved from the eukaryotic *SNF2* QLQ domain, most likely by a duplication event in the common ancestor of the Charophytes and land plants. Altogether, our results are important for advancing the origin and evolution of the GRF family in Streptophyta.

## Introduction

Growth Regulating Factors (GRFs) compose an important transcription factor family that plays diverse roles in plant development. These transcription factors are characterized by the obligatory presence of 2 conserved domains named QLQ (Gln, Leu, Gln) and WRC (Trp, Arg, Cys) (van der Knaap *et al.*, 2000). The QLQ domain is usually located at the protein N-terminus and contains the motif QX_3_LX_2_Q. This region is named QLQ due to the similarity to the protein-protein interaction domain of the yeast SWI2/SNF2 (Switch/Sucrose non-fermentable), which is a subunit of a chromatin-remodeling complex (van der Knaap *et al.*, 2000). Located after the QLQ, the WRC domain contains a nuclear localization signal and a CX_9_CX_10_CX_2_H motif (van der Knaap *et al.*, 2000), which is an atypical C3H Zinc-finger motif found in barley HRT (Hordeum repressor of transcription), a transcriptional repressor of the Gibberellin Response Element (GARE) (Raventós *et al.*, 1998). Further studies have demonstrated that the WRC domain from GRFs acts as DNA binding domain in barley, Arabidopsis, and rice (Osnato *et al.*, 2010; Kim *et al.*, 2012; Kuijt *et al.*, 2014), and that some GRFs possess more than one WRC, such as AtGRF9 (Kim *et al.*, 2003) and BrGRF12 (Wang *et al.*, 2014). Besides that, there are other conserved regions found in the C-termini of some but not all GRFs, such as FFD (Phe, Phe, Asp), TQL (Thr, Gln, Leu), and GGPL (Gly, Gly, Pro, Leu) (van der Knaap *et al.*, 2000; Kim *et al.*, 2003; Zhang *et al.*, 2008); however, their roles were not yet unveiled (Kim and Tsukaya, 2015).

Most of the studies in recent years have focused on the understanding of the specific roles of GRFs in different plant species (Omidbakhshfard *et al.*, 2015; Kim and Tsukaya, 2015). The first known functions described for these proteins were in stem and leaf growth, particularly in GA-induced stem elongation (van der Knaap *et al.*, 2000), regulation of cell proliferation in leaf primordia (Horiguchi *et al.*, 2005; Kim and Lee, 2006), cotyledons and shoot apical meristem (SAM) (Kim and Lee, 2006; Kuijt *et al.*, 2014). Other functions related to plant development were also revealed, including participation in flower organogenesis (Liu *et al.*, 2014), organ longevity (Debernardi *et al.*, 2014; Vercruyssen *et al.*, 2015), seed oil production (Liu *et al.*, 2012), photosynthetic efficiency (Liu *et al.*, 2012; Vercruyssen *et al.*, 2015), control of grain size and yield (Che *et al.*, 2015; Duan *et al.*, 2015; Hu *et al.*, 2015; Li *et al.*, 2016; Sun *et al.*, 2016). Importantly, GRF genes are known to be upstream regulators of class I KNOX (KNOTTED1-like homeobox) genes required to maintain an appropriate level of SAM activity, together with other regulators of KNOX I expression, and this function is conserved in monocot and eudicot species (Kuijt *et al.*, 2014; Tsuda and Hake, 2015). Under adverse environmental conditions, GRFs also play important roles, such as coordination of growth in response to osmotic and ABA-induced stresses (Kim *et al.*, 2012) and host transcriptional reprogramming during cyst nematode infection (Hewezi *et al.*, 2012) and in response to fungal pathogens (Soto-Suárez *et al.*, 2017).

GRFs can physically interact with GRF-Interacting Factors (GIFs), a small family of transcriptional co-activators. This interaction occurs between the QLQ domain of GRF and the SNH (SSXT N-terminal homolog) domain present in GIF proteins (Kim and Kende, 2004). However, this interaction does not seem to be mandatory for GRF function because GRFs are capable of acting as negative regulators (Kim *et al.*, 2012; Kuijt *et al.*, 2014). Recently, it was demonstrated that the functioning of the GRF-GIF duo may be associated with the auxin signaling network (Lee *et al.*, 2018). Also, it is not clear whether distinctive heterodimers of GRF and GIF have different functions in the downstream pathways (Kim and Tsukaya, 2015).

GRFs are part of a complex regulatory module. Some GRF members are negatively regulated at the transcript level by miR396 (Rodriguez *et al.*, 2010; Wang *et al.*, 2011; Hewezi *et al.*, 2012; Debernardi *et al.*, 2014). The miRNA396 responds to different stress conditions such as drought, cold, high-salinity, UV-B light, and pathogens (Liu *et al.*, 2008; Zhou *et al.*, 2012; Casadevall *et al.*, 2013; Soto-Suárez *et al.*, 2017), and it is also regulated by the TCP family (TEOSINTE BRANCHED1, CYCLOIDEA, and PROLIFERATING CELL NUCLEAR ANTIGEN FACTOR1) (Schommer *et al.*, 2014), which also modulates the gene expression of GRFs and GIFs directly (Rodriguez *et al.*, 2010). Moreover, GRFs affect miR396 transcript levels and then, the gene expression of other GRFs (Hewezi *et al.*, 2012), in an intricate cascade of regulation.

In Arabidopsis, GIF1, also called ANGUSTIFOLIA3 (AN3), is a homolog to the human Synovial Translocation Protein (SYT) (Kim and Kende, 2004). Interestingly, SYT interacts with the human SNF2 proteins, BRM (Brahma), and BRG (Brahma-related gene 1) (Nagai *et al.*, 2001). Also, in Arabidopsis, GIF1 can associate with 2 different SWI/SNF complexes through the interaction with BRM or SYD (Splayed), the SNF2 homologs in this species (Debernardi *et al.*, 2014).

SNF2 protein is part of a homonymous subfamily of the SNF2 family. Whereas the SNF2 family is characterized by the presence of a conserved SNF2 domain, QLQ is found only in the SNF2 subfamily (Eisen *et al.*, 1995; Ryan and Owen-Hughes, 2011). Although the SNF2 and GRF proteins are known to share a conserved QLQ domain located at the *N-termini* of both proteins, and have the same molecular partner GIF or its ortholog SYT, to date, there has been no study addressing the evolutionary aspects related to the origin of the GRFs or exploring the divergence between GRF and SNF2.

GRF-encoding genes are found in plant genomes, including the Charophyte *Klesormidium nitens* (Kim and Tsukaya, 2015; Omidbakhshfard *et al.*, 2015; Cao *et al.*, 2016; Catarino *et al.*, 2016; Wilhelmsson *et al.*, 2017), suggesting that the emergence of this transcription factor may precede the occurrence of the land plants. Based on phylogenetic analysis, previous studies proposed divisions of *GRFs* in six (Omidbakhshfard *et al.*, 2015) or five (Cao *et al.*, 2016) groups. The former study claims that the GRF genes evolved via an eudicot whole-genome triplication and other independent WGD events, followed by gene retention in the ancestors of soybean and poplar Among the 6 groups, the authors found two groups specific to eudicot species and no group exclusive to monocots (Omidbakhshfard *et al.*, 2015). The latter study focused on Arabidopsis, rice, Chinese pear, poplar, and grape genes. Among the five groups, three contain genes from the five species, whereas the other two groups include genes from one, two, or three species. Also, they found one group specific to monocots and one exclusive to eudicot species (Cao *et al.*, 2016).

Many aspects of the biological functions of GRFs are already well known. However, the evolutionary history and diversification of these proteins are not yet completely elucidated and need to be more deeply comprehended based on different methods and discussed in detail. In this work, we conducted a phylogenetic approach to understand the evolution and diversification of the *GRF* gene family.

Based on the divergence within the QLQ domain found in SNF2 and GRF and on the distribution of each family across distinct taxa, we hypothesize that GRFs evolved from SNF2 and were established as a new transcription factor in the common ancestor of the Charophytes and land plants. In addition, we suggest that SNF2 and GRFs’ QLQ domains diverged particularly early in the course of evolution, most likely as a result of a duplication event. Also, we found well-supported data for eleven groups of GRF genes in flowering plants, six groups exclusive to eudicots, and five groups exclusive to monocot species, suggesting that the GRF family evolved mostly independently in monocot and eudicot species.

## Material and Methods

### Sequence retrieval

The sequences were retrieved from the public databases Phytozome v12.0 (Goodstein *et al.*, 2012) (www.phytozome.jgi.doe.gov/pz/portal.html), Metazome v3.2 (available at www.metazome.jgi.doe.gov/pz/portal.html), NCBI (https://blast.ncbi.nlm.nih.gov/Blast.cgi), FernBase (https://www.fernbase.org/), Congenie (http://congenie.org/), MarpolBase (http://marchantia.info/), and K*lebsormidium nitens* NIES_2285 genome project v1.1 (Hori *et al.*, 2014) (available at: www.plantmorphogenesis.bio.titech.ac.jp/~algae_genome_project/klebsormidium). A detailed list of all species and *loci* used in this work is provided in Tables S1, S2, and S3.

For GRF sequences, two previously identified sequences - OsGRF1 (van der Knaap *et al.*, 2000) and AtGRF1 (Kim *et al.*, 2003) - were used as queries in blastp, besides searches for QLQ and WRC annotated domains in the Phytozome database. The searches were conducted against 40 sequenced plant genomes (Table S2) and four Chlorophytes (green macroalgae) genomes (*Chlamydomonas reinhardtii*, *Volvox carteri*, *Micromonas sp*. RCC299 and *Ostreococcus lucimarinus*), available at Phytozome. The charophytes are the extant group of green algae that are most closely related to modern land plants. We conducted a blast search against the Charophyte species K*lebsormidium nitens* NIES 2285 genome to check the presence of *GRFs* in this organism.

**Figure 1:**
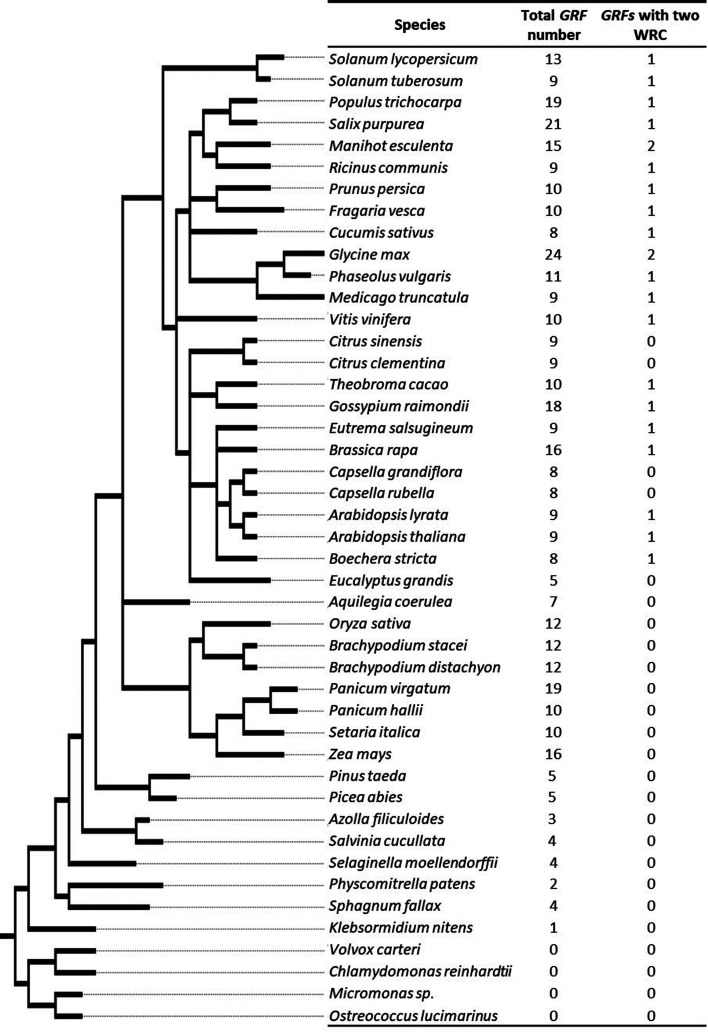
Tree of species. Species searched and the number of genes found in each species. The tree was based on NCBI Taxonomy and was constructed with phyloT, available at: http://phylot.biobyte.de

A tree of the 45 species was reconstructed with phyloT (available at http://phylot.biobyte.de) to facilitate the visualization of GRF expansion in different species (Figure 1). Because *K. nitens* is a unique Charophyta alga with a sequenced genome available, we performed blast searches using transcriptomic data from *Spirogyra pratensis*, *Nitella mirabilis*, *Mesostigma viride*, *Closterium peracerosum-strigosum-littorale*, and *Klebsormidium crenulatum.* The blast search was conducted using the GRF sequence from *K. nitens* as query. The retrieved sequences were analyzed in ScanProsite (Castro *et al.*, 2006) to verify the presence of both QLQ and WRC domains. Complete protein sequences were subjected to domain analysis, and only sequences presenting both domains were considered to be GRFs. We found GRFs only in Charophyta and land plants. From 415 GRF sequences, three were discarded from the phylogenies due to low-score domains or bad-quality alignments (Table S2).

For SNF2 analysis, SNF2 from *Saccharomyces cerevisiae* (NM_001183709) was used as the query for blastp in the NCBI database against nine fungi species and in the Metazome against 11 complete sequenced genomes. Plant and *K. nitens* sequences were searched using the SNF2-related BRAHMA (BRM) from Arabidopsis as the query in blastp against the genomes of seven plant and five algae species in Phytozome and *K. nitens* genomes (Table S1).

### Sequence alignments and evolutionary analyses

Sequence alignments were performed using CDS sequences from QLQ and WRC considering codon position, using the MUSCLE algorithm (Edgar, 2004), available at MEGA 7.0 (Molecular Evolutionary Genetics Analysis) (Kumar *et al.*, 2016). The sequences were checked to find QLQ and WRC domains, which were used for phylogenetic analysis. Phylogenetic trees were reconstructed using nucleotide sequences of QLQ and WRC domains by Bayesian inference using BEAST2.4.5 (Bouckaert *et al.*, 2014).

For GRF sequences, the best fit model for nucleotide evolution was GTR with invariable sites and gamma-distributed rates. A smaller tree containing sequences from Arabidopsis, rice, and the moss *P. patens* was reconstructed with the same parameters to allow a better understanding of the gene structure analysis of these species. For SNF2-GRF analysis, the best fit model for nucleotide evolution was TPM2 with invariable sites and gamma-distributed rates. Both models were selected with jModeltest v2.1.7 (http://jmodeltest.org/). The Birth and Death Model was selected as tree prior, and 100,000,000 generations were performed with Markov Chain Monte Carlo algorithm (MCMC) (Gilks, 2005) for evaluation of posterior distributions in all cases.

After manual inspection of the alignments, 415 sequences were used based on alignment quality and the presence of both QLQ and WRC domains for GRF analysis, totaling 243 DNA sites, 108 corresponding to QLQ, and 135 corresponding to WRC. For SNF2-GRF analysis, 131 sequences from QLQ domains with 108 DNA sites were used. In both cases, convergence was verified with Tracer v.1.6 (Rambaut *et al.*, 2014) (http://beast.bio.ed.ac.uk/Tracer), and consensus trees were generated using TreeAnnotator, available at BEAST package. The resulting trees were viewed and edited using FigTree v.1.4.3.

Using GRF-SNF2 alignment and the respective phylogenetic tree as input, the rates of nonsynonymous to synonymous substitutions (dN/dS or ω) were computed, and homogeneity and positive selection were determined using maximum-likelihood models in the program CODEML in PAML (v.4.9) (Yang, 2007). For site model analysis, models M0 (basic), M1 (nearly neutral), M2 (selection), M3 (discrete), M7 (beta distribution, ω > 1 disallowed), and M8 (beta distribution, ω > 1 allowed) were considered (Goldman and Yang, 1994; Yang and Nielsen, 1998; Yang, 2000; Yang *et al.*, 2005). The branch-site model was carried out comparing the alternative model (model = 2, Nsites = 2, fix_omega = 0, and omega = 0) with its null model (model = 2, Nsites = 2, fix_omega = 1, and omega = 1) (Zhang *et al.*, 2005; Yang and Reis, 2011). The GRF branch was selected as the foreground, and statistical significance was addressed using LRT. CodeML was set to estimate branch lengths by using random starting points (fix_blenght = -1) and the F3x4 option for expected codon frequencies based on 3-codon positions. Naive empirical Bayes and Bayes empirical Bayes approaches were used to calculate the posterior probability of each site within the alternative model.

### Domain architecture and gene structure analysis

Complete protein sequences of 392 GRFs were submitted to MEME Suite v4.12.0 (Bailey *et al.*, 2009) (http://meme-suite.org/) to search for five different motifs in any number of repetitions, in order to find different combinations of QLQ, WRC, FFD, TQL, and GGPL in GRF proteins. We set a cut-off E-value of 10^-6^ to avoid false positives. The specific positions of the domains were used to construct a diagram presented in Figure S1. Protein sequences corresponding to the domains of all genes used in phylogeny analysis were used to construct the logos of the five domains on WebLogo3 (Crooks *et al.*, 2004). For gene structure analysis, we used genomic sequences of three representative species, Arabidopsis, rice, and *P. patens*. The information about intron/exon organization was retrieved from Phytozome.

### Synteny analysis and chromosomal locations

To better understand the pattern of expansion of GRFs, we conducted synteny analysis on PLAZA 4.0 (Van Bel *et al.*, 2018). Synteny is based on the occurrence of collinear blocks between genomes, and these blocks are identified by the presence of homolog genes, also referred to as anchors, in both genomes or in different segments inside a genome.

The loci of GRFs from Arabidopsis, soybean, tomato, rice, maize, and purple false brome were searched in PLAZA 4.0 to find anchor points between different GRFs. The synteny relationships between the genomes were illustrated using CIRCOS (Krzywinski *et al.*, 2009). The chromosomal positions and duplications of Arabidopsis and rice GRFs were drawn from information obtained from NCBI and PLAZA 4.0 databases, respectively.

### Identification of OsGRF putative targets

To identify putative targets of the rice GRFs, we determined the location of the conserved motif “TGTCAG” or the reverse complement “CTGACA” in the rice genome using the fuzznuc tool from EMBOSS (Rice *et al.*, 2000). All the hits were annotated back in the rice genome using the ChIPpeakAnno package (Zhu *et al.*, 2010) for the R environment. Genes containing at least two motifs within 1500 bp upstream of ATG were selected using a customized R script. The functional annotation of Gene Ontology terms and a statistical overrepresentation test were performed using the PANTHER 11 (Mi *et al.*, 2017) database with default settings, and only results with P&lt;0.05 were considered.

## Results

### Identification of GRF genes and QLQ divergence from SNF2

We analyzed 45 plant genomes and found *GRF* genes in 41 of them. Viridiplantae separated into Chlorophyta and Streptophyta approximately 629 to 890 million years ago (Morris *et al.*, 2018). Streptophyta comprises Embryophyta, referred to as “land plants”, and six distinct groups of Charophyte algae: Mesostigmales, Chlorkybales, Klebsormdiales, Charales, Coleochaetales, and Zygnematales.

We also found a GRF gene in the genome of the Charophyte algae *Klebsormidium nitens* (formerly *Klebsormidium flaccidum*). As previously reported, we did not find *GRFs* in Chlorophytes. A total of 410 GRF-encoding genes were identified, of which 22 produce proteins containing 2 WRC domains (Figure 1).

In addition to the GRF genes previously described (Zhang *et al.*, 2008; Filiz *et al.*, 2014; Cao *et al.*, 2016), we found four extra genes in the maize genome, *ZmGRF15* to *18*. We discarded *ZmGRF8* and *12* from our analyses because the former contains only a partial WRC domain and does not have QLQ, and in the latter one, both domains are absent; however, we kept the nomenclature to avoid future confusion. We also found two additional genes in purple false brome (*BdiGRF11* and *12*) and two extra genes in grapevine (*VviGRF9 and VviGRF10*) (Tables S2 and S3).

We also analyzed the divergence between *GRF* and *SNF2* because both share a conserved QLQ domain, which allows the interaction with SNH domains present in the homologous SYT and GIF families. Whereas GRFs are exclusive to Streptophyta, *SNF2* genes are widely present in eukaryotes, such as fungi, metazoan, and plants, and compose a subfamily of the SNF2 family (Eisen *et al.*, 1995; Ryan and Owen-Hughes, 2011). The SNF2 subfamily genes are the only representatives of the SNF2 family that have a QLQ domain. These genes have different names, such as *SNF2* in fungi, *BRM* and *BRG1* in metazoans, and *BRM* and *SPLAYED* (*SYD*) in plants. In this work, the general term “*SNF2*” was used for all of the *SNF2*-type genes. However, when referring to a particular gene, the specific gene name was used.

The binding region between BRM (the human SNF2) and SYT (the GIF homolog) was shown to be located between the amino acids 156 to 205 for BRM, and 1 to 181 for SYT (Nagai *et al.*, 2001). Analyses in SMART (Simple Modular Architecture Tool) (Letunic *et al.*, 2015) showed that these regions correspond to QLQ (172 to 208) and SNH (17 to 77) domains, respectively. The interaction between GRFs and GIFs also occurs via these domains (Kim and Kende, 2004). From blastp analyses, we were able to identify SNF2 homologs in fungi, metazoans, algae, and plants. Fifty-two encoding genes from 32 species were further selected for our analysis (Table S1).

**Figure 2:**
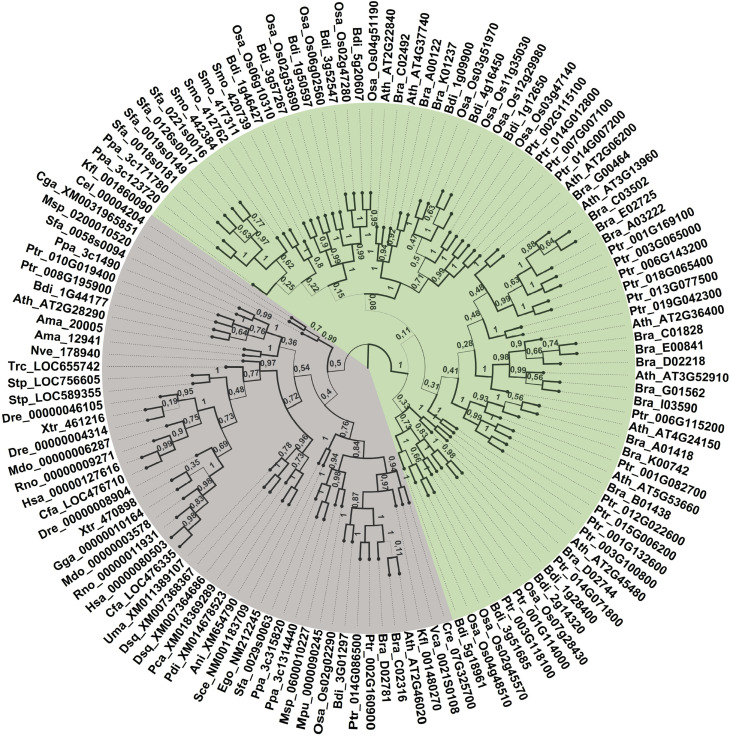
Phylogenetic relationship among QLQ from *SNF2* and *GRFs*. The tree with SNF2- and GRF-coding sequences of QLQ domains of algae, land plants, animals, and fungi was reconstructed by Bayesian inference. *GRFs* are colored in green, *SNFs* are colored in gray. The species and *loci* information are detailed in Table S1.

The phylogenetic relationships of *SNF2* and *GRF* gene families revealed that *GRFs* and *SNF2* grouped in distinct clades. While all GRFs are grouped in a well-supported cluster, SNF2 members are organized into smaller groups. Higher divergence within the QLQ domain found in SNF2 is consistent with its prevalence across distant taxa because it is present in diverse eukaryotic species. Also, the extent of conservation within the QLQ domain that comprises GRFs transcription factors stems from these proteins having evolved more recently (Figures 2 and 6).

**Figure 3:**
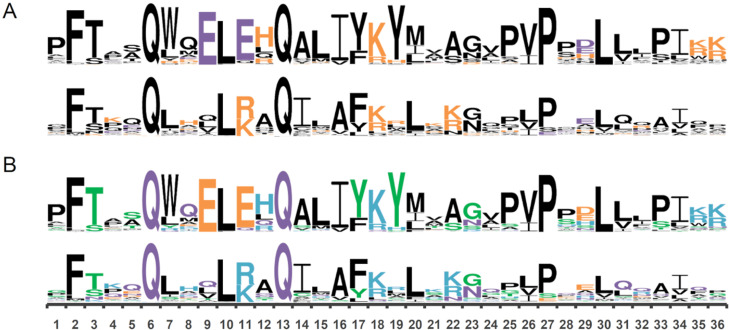
QLQ properties in SNF2 and GRF. (A) Logos representing the charge of the amino acids from the QLQ domain present in GRF (top) and SNF2 (bottom). Negative residues in purple and positive in orange. (B) Logos representing chemical properties of the amino acids from QLQ domain of GRF (top) and SNF2 (bottom). Basic residues in blue, acidic in orange, neutral in purple, polar in green, and hydrophobic in black. Protein sequences corresponding to the QLQ domain from SNF2 and GRFs were used separately to construct the diagrams in WebLogo3.

**Figure 4:**
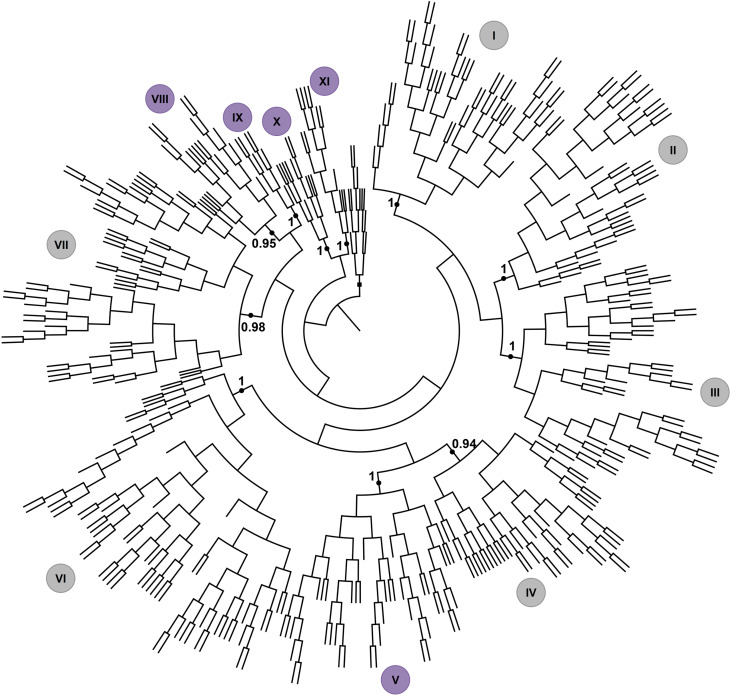
Phylogenetic relationship of *GRFs.* Coding sequences of QLQ and WRC domains of *K. nitens* and land plants were used to generate a phylogenetic tree reconstructed by Bayesian inference. Groups I to XI correspond to flowering plants, and the others correspond to algae and moss sequences. The dicot groups are colored in gray, whereas the monocot groups are colored in purple. Species, *loci*, and taxa terminologies are available in Table S2. A detailed tree is available in Figure S1.

**Figure 5:**
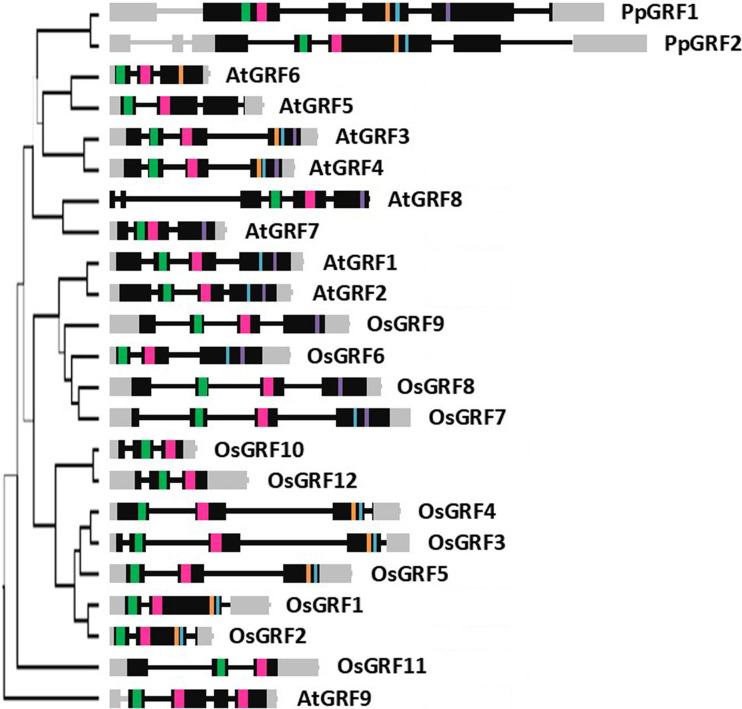
Structural organization of *GRF* genes. QLQ and WRC coding sequences of GRFs of *P. patens* (Pp), *A. thaliana* (At), and *O. sativa* (Os) were used to generate a phylogenetic tree by Bayesian inference (line width by posterior probability). The graphical representation of gene structures was based on genomic information available at Phytozome. Gray color corresponds to 5’ and 3’ untranslated regions. Black bars and lines indicate exons and introns, respectively. The different domains are colored in green (QLQ), pink (WRC), orange (TQL), blue (TQL), and purple (GGPL). For scale, QLQ domain (green) corresponds to 118bp.

**Figure 6:**
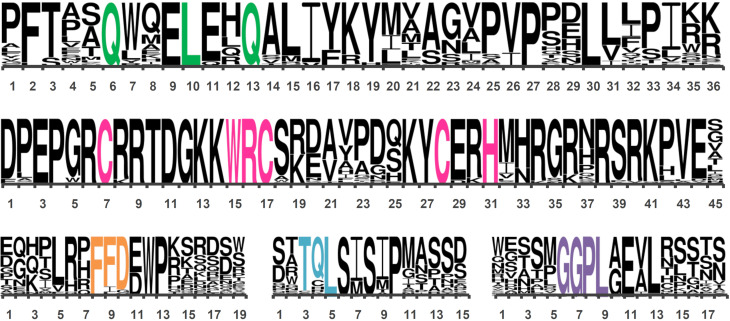
Analysis of GRF motif conservation. Logos of QLQ (36 aa), WRC with the C3H motif (45 aa), FFD (19 aa), TQL (15 aa), and GGPL (18 aa) domains. The height of the letters is based on the probability of the residue in the position, and the width was adjusted to fit. Domain colors: QLQ in green, WRC in pink, FFD in orange, TQL in blue, and GGPL in purple.

*AtBRM* and *AtSYD* are paralogous that grouped into distinct subclades in the *SNF2* clade. In addition to Arabidopsis, purple false brome, turnip, and populus possess both genes, whereas rice and turnip have only *BRM*. Both *BRM* and *SYD* suffered specific duplications in populus, and *BRM* was duplicated in turnip, probably in a WGD event. The detailed information on species, *loci*, and taxa terminologies of *SNF2* and *GRFs* are provided in Table S1 and S2, respectively.

The early divergence between the QLQ domains of *SNF2* and *GRFs* was accompanied by changes in the amino acid composition and therefore in the properties of QLQ domain. In GRF proteins, QLQ domain presents two conserved glutamic acid (E) residues in positions 9 and 11, conferring a negative charge and acidic property to the core. In the case of SNF2, the charge is neutral to positive, and the chemical property varies from neutral to basic in the same positions (Figure 3A and 3B). Other prominent differences are observed in positions 12, 22, 35, and 36. Besides the canonical QX_3_LX_2_Q, the most conserved residues are the phenylalanine (F) in position 2, the proline (P) in position 27, and the leucine (L) at position 30 (Figure 3A and 3B).

To analyze which protein sites might be under positive selection, we performed site model and branch-site model analyses. The site model assumes that some sites are under positive selection on all tree branches, whereas the branch-site model assumes that positive selection may be taking place on the foreground branch only. The site model analyses of the SNF2-GRF group revealed significant evolutionary constraints in the QLQ domain. The log-likelihood difference between models M0 and M3 was statistically different (Table S4), suggesting that ω is heterogenous among the analyzed sites; however, positive selection was not detected through this approach. On the other hand, branch-site model analysis revealed positive selection for the branch leading to GRF (Table S4). When comparing GRF, defined as the foreground, with the SNF2 representatives, it was possible to detect positive selection at positions 11, 12, and 22, which are associated with significant changes within the QLQ domain. Positive selection was also detected at QLQ positions 7, 16, 19, 24, 31, 32, and 34.

### Diversification of GRF family in Streptophyta

**Figure 7:**
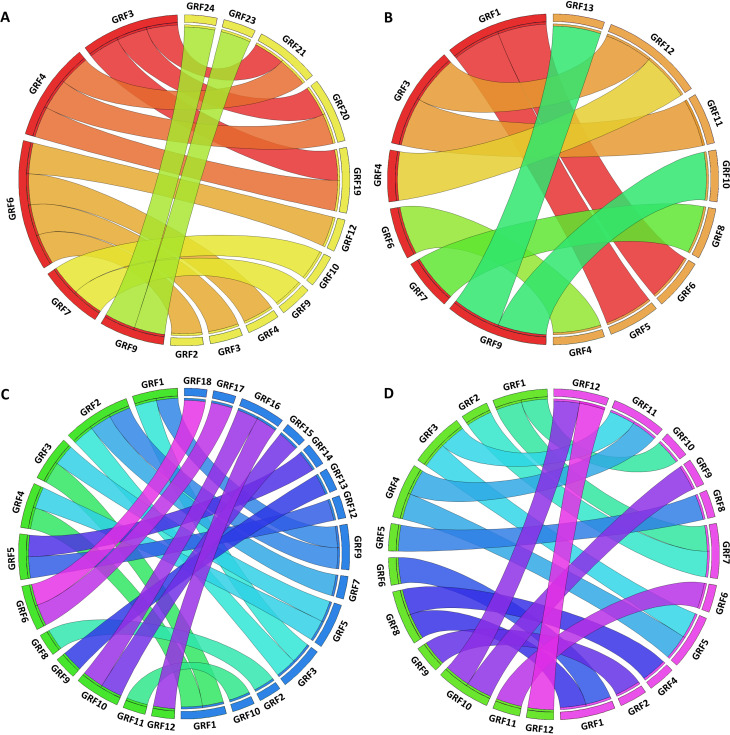
Syntenic analysis of *GRFs* in different species. Synteny between (A) Arabidopsis (red) and soybean (yellow), (B) Arabidopsis (red) and tomato (orange); (C) rice (green) and maize (blue), (D) rice (green) and purple false brome (purple). Probable orthologs are linked by ribbons.

The *GRF* family has undergone a significant expansion in land plants. The phylogenetic tree, reconstructed from 392 sequences by the Bayesian method, allowed us to identify 11 well-supported groups of GRF proteins in flowering plants, as shown by the posterior probability (Figure 4 and Figure S1). The composition of the groups is consistent with domain distribution in all GRF proteins (Figure S1), and with gene structural organization (Figure 5) and synteny analysis (Figure 7A and 7B) from selected species.

The expansion of the GRF gene family occurred independently for the current monocot and eudicot species. There are six groups exclusive to eudicots: groups I to IV, VI and VII; and five groups exclusive to monocots: groups V and VIII to XI. For both monocots and eudicots, duplication events occurred mainly on the basis of each one of these groups of species because the genes from different species are almost ubiquitous throughout each group. A phylogenetic tree showing the separation of the 11 groups is provided in Figure 4, and the complete tree containing all *taxa* terminologies, group relationships, and domain characterization of all GRFs is found in Figure S1. Sequences derived from the *Klebsormidium* genome and from bryophytes and lycophytes representatives did not cluster in well-supported groups in our analysis (Figure 4).

To gain further insight into GRF diversification, sequences from fern (*Azolla Filiculoides* and *Salvinia cucullata*) and Gymnosperm (*Picea abies* and *Pinus taeda*) genomes, as well as from *Spirogyra pratensis*, *Nitella mirabilis*, *Mesostigma viride*, *Closterium peracerosum-strigosum-littorale*, and *Klebsormidium crenulatum* transcriptomes, were added to the analysis. This second analysis recovered the same 11 well-supported groups than the previous analysis (Figure S2), for which reason we deepened our discussion within these groups.

In general, Group I is characterized by the presence of proteins containing five domains. A duplication in the basis of Brassicaceae originated *AtGRF3* and *AtGRF4* and their orthologs. Duplications in the basis of eudicots were responsible for the Group II expansion, giving rise to a subgroup of 15 genes encoding proteins containing one WRC domain and 22 GRFs presenting 2 WRC domains. In most cases, there is no additional motif, with some exceptions where TQL is present.

Group III is characterized by the presence of the GGPL, which corresponds to the only additional domain, and basal duplications gave rise to subgroups containing *AtGRF7* and *AtGRF8*. Proteins from Groups IV and V have similar structures, with the presence of FFD and TQL additional domains. Group IV is exclusive to eudicots, whereas group V comprises GRFs from monocot species. The similarity between these groups may be explained by a common ancestor gene that evolved independently in monocot and dicot species. The expansion of group IV occurred on the basis of eudicots, and the Brassicaceae ancestor probably suffered gene loss because there are no members in this group. The expansion of the Group V occurred mainly via duplications in the origins of Poaceae, originating 5 subgroups. These duplications gave rise to the paralogous *OsGRF1* and *OsGRF2*, *OsGRF3* and *OsGRF4*, and the closely related gene *OsGRF5*.

Group VI is exclusive to eudicots and present subgroups containing different extra domains. The subset containing *AtGRF5* and *AtGRF6* is specific to Brassicaceae. The first possess only QLQ and WRC, and the second also contains the FFD domain. Although this group has a diversified protein structure, *AtGRF5* and *AtGRF6* are syntenic to other genes present in this group. Group VII arose in the basis of eudicots. In general, members of this group possess TQL and GGPL, with some exceptions. Duplication in the basis of Brassicaceae gave rise to the paralogous *AtGRF1* and *AtGRF2*, presenting TQL and GGPL.

Groups VIII, IX, X, and XI evolved from an ancestor of Poaceae. Groups VIII and IX are more related to the eudicot Group VII and may have a common ancestor gene that diverged independently in monocot and dicot species. A basal duplication in Group VIII originated 2 subgroups, the first containing *OsGRF6* and its orthologs, possessing TQL and GGPL, and the other subgroup containing *OsGRF7*, *OsGRF8*, and its orthologs, with the GGPL domain. Group IX, in which *OsGRF9* is present, originated in the Poaceae ancestor and has only a GGPL extra domain.

Groups X and XI probably evolved with basal duplications. The structure of the members of these groups is formed by QLQ and WRC only, without the presence of additional domains. Group X is formed by *OsGRF11*, *ZmGRF10*, and other genes, whereas group XI comprises *OsGRF10* and *OsGRF12*, among others. Also, the *GRFs* in these groups have an extremely short C-terminal region and the absence of additional domains. Despite the similarity between these groups, the low posterior probability in the consensus tree did not support a single clade between the groups X and XI, suggesting the existence of some level of divergence between them.

Structural organization of GRF genes from Arabidopsis, rice, and moss

We selected Arabidopsis, rice, and *moss* as representative species of eudicots, monocots and mosses to analyze the structural organization of *GRF* genes between these clades. Among these three species, the two *GRF* genes from the moss *Physcomitrella patens* are the largest, with 6200 and 6399 bp, respectively. *OsGRFs* ranged from 1126 to 3948 bp and *AtGRFs* from 1053 to 3416 bp. To facilitate comparison of gene structures, a tree was reconstructed with sequences of only these three species (Figure 5). Most of the genes have QLQ and WRC in separate exons, except for *PpGRF1* and *AtGRF7*. The number of introns interrupting the coding region varied from 2 to 4, and domain position follows the order QLQ, WRC, FFD, TQL, and then GGPL.

In general, genes positioned in the same group have highly similar gene structures, besides domain composition. The two PpGRFs have 4 introns interrupting the coding region. AtGRF5 and 6, both from Group VI have a similar organization; however, *AtGRF5* lost the FFD domain. From Group I, both *AtGRF3* and *AtGRF4* have 4 exons and 3 introns interrupting the coding region and possess all the 5 domains. *AtGRF7* and *8*, from Group III, have GGPL as the only extra domain but present different genetic structures. *AtGRF1* and *AtGRF2*, from Group VII, both possess 4 exons and 3 introns, with TQL and GGPL in the last exon. *OsGRF9* from Group IX has 3 introns, 4 exons, and a GGPL domain. From Group VIII, *OsGRF6* have 2 introns and 3 exons, and the closely related *OsGRF7* and *OsGRF8* have 3 introns and 4 exons. *OsGRF10* and *OsGRF12*, both from Group XI, have 2 introns and 3 exons, and no additional domain. *OsGRF1* to *5*, from Group V, have similar gene structures, with the presence of FFD and TQL domains. The subgroup including *OsGRF1* and *OsGRF2* contains 3 exons and 2 introns, whereas the subgroup of *OsGRF3* to *5* has an additional intron separating WRC from the extra domains. *OsGRF11*, from Group X, have no additional domain, and *AtGRF9*, from Group II, possess an extra WRC motif.

### Domain conservation of GRFs

We also analyzed the amino acid sequence conservation of the five domains in 392 GRF sequences to identify the pattern of conservation and the polymorphic sites (Figure 6). Among the five domains, WRC is the most conserved, except for the region between the positions 19 to 25 that is less conserved. We found an absolute conservation of the C3H motif, suggesting the importance of this motif for GRF function. QLQ domain has some sites with high conservation, importantly, the QX3LX2Q, the phenylalanine (F) in position 2, 2 glutamic acid (E) residues in positions 9 and 11, the proline (P) in position 27, and the leucine (L) in position 30, among others. FFD have a higher conservation in the core of the motif. Besides 2 phenylalanine (F) and the aspartic acid (D) residues in positions 8 to 10, this domain possesses tryptophan (W) in position 12, and proline (P) in 13. TQL has three sites even more conserved than the amino acids present in positions 3 to 5 that appoint the domain. Two serine (S) and one proline (P) residues, localized in the sites 6, 8, and 10 respectively, are almost absolutely conserved. The GGPL domain also has core conservations, with glutamic acid (E) and leucine (L) in positions 11 and 13, besides the two glycines (G), the proline (P), and the leucine (L) that names the motif, located at positions 6 to 9.

### Synteny analysis and genomic organization of GRF genes

To find probable orthologs of *AtGRFs* and *OsGRFs*, we conducted searches in PLAZA 4.0 (Van Bel *et al.*, 2018). Arabidopsis *GRFs* were searched against tomato and soybean genomes, and rice *GRFs* were searched against maize and purple false brome genomes. The pairs of probable orthologs found are summarized in Figure 7. In general, these pairs are consistent with the distribution of the genes in the groups of the phylogenetic tree.

**Figure 8:**
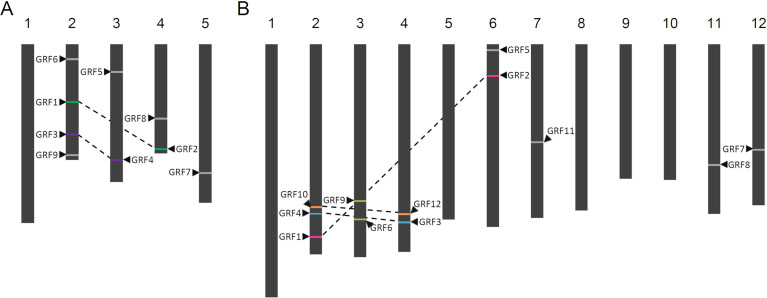
Distribution of GRFs in nuclear chromosomes of Arabidopsis and rice**.** The positions of the GRFs from (A) Arabidopsis and (B) rice are indicated by arrows. Genes in duplicated regions are represented with the same colors. Genes not duplicated are represented in gray. Duplicated regions in different chromosomes are linked with dashed lines.

We also analyzed the intraspecific duplications of GRFs in Arabidopsis and rice using the same database. The relative positions of the genes and the duplicated blocks are graphically displayed in Figure 8. *AtGRF1* and *AtGRF2* are located on chromosomes 2 and 4, respectively. Both genes are members of Group VII. Group I members are *AtGRF3*, located on chromosome 2, and *AtGRF4*, located on chromosome 3. *OsGRF1* and *OsGRF2* are located on chromosomes 2 and 6. *OsGRF3* and *OsGRF4* are located on chromosomes 4 and 2. These latter 4 genes are members of Group V, and the syntenic genes form different subsets inside the main group. *OsGRF6* and *OsGRF9* are both located on chromosome 3.

### *In silico* prediction of GRF targets and biological processes

A target *cis*-element for *AtGRF6* and *7* transcription factors were characterized by functional (Kim *et al.*, 2012) and cistrome (O’Malley *et al.*, 2016) analyses. The regulatory sequences described in these previous works present the core nucleotides “TGTCAG” that was first discovered in the *DREB2A* promoter, which is regulated by AtGRF7 (Kim *et al.*, 2012). In rice, one study showed that GRF binding activity to the promoter of the KNOX gene *Oskn2* was associated with the presence of CTG or CAG repeats (Kuijt *et al.*, 2014). It is not known whether these target sequences are conserved among different species; however, one hypothesis for the maintenance of multiple binding sites is that it contributes to the regulation of a plethora of genes.

Initial studies from our group suggest the functionality of “TGTCAG” in the regulation of *OsGRF11* targets in rice (Fonini, 2017). Here, we conducted an *in silico* analysis to find putative targets of GRFs by the *cis*-element core “TGTCAG” or the reverse complement “CTGACA” in this species, whereas the CTG or CAG repeats are not suitable for this type of analysis.

The identification of putative targets was conducted in the *fuzznuc* tool from EMBOSS (Rice *et al.*, 2000). We identified genes containing at least two core motifs in a region of 1,500 bp upstream of ATG. A set list containing 1270 putative targets of GRFs was submitted to Gene Ontology analysis and an overrepresentation test in the PANTHER (Mi *et al.*, 2017) database. From these, 83 were not annotated in the database, and seven had multiple mapping information. The complete list of enriched GO terms and the 1270 putative targets are available in Tables S5 and S6, respectively. The statistic overrepresentation test demonstrates that the enriched targets are involved in several biological processes related to GRF functions. Among these processes are the regulation of leaf development (GO:2000024), regulation of endosperm development (GO:2000014), adaxial/abaxial pattern formation (GO:2000011), regulation of meristem structural organization (GO:0009934), reproductive process (GO:0022414), cell cycle (GO:0007049), cell division (GO:0051301), and regulation of cell proliferation (GO:0042127).

This result suggests that the *cis*-element is conserved (at least in rice) because several biological processes of the putative targets match with already-characterized GRF functions. Also, this target library may contribute to functional GRF studies in rice and in other species.

## Discussion

**Figure 9:**
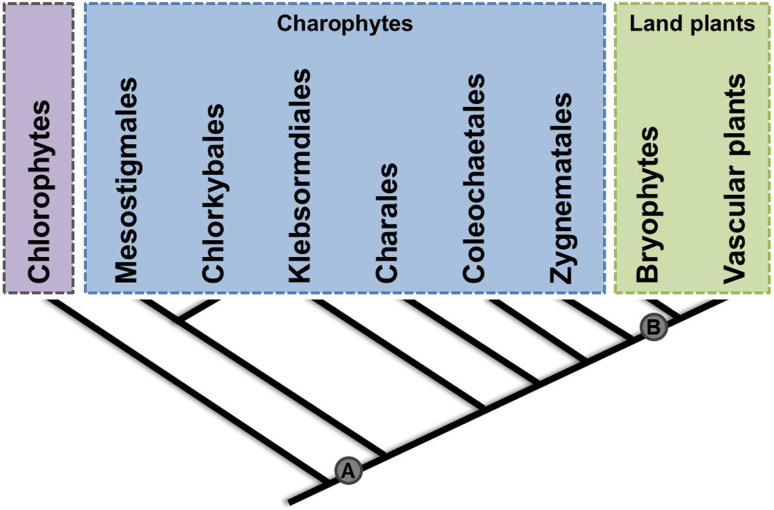
Emergence and expansion of *GRF* family in Viridiplantae. (A) *GRF* emerged in a charophyta ancestor previous to Mesostigmales. (B) The expansion of the *GRF* family occurred in land plants. Diagram of the relationships between algae and plant lineages is based on data already published (Morris *et al.*, 2018).

In this work, we analyzed 45 plant and algal genomes and reconstructed the evolutionary history of the GRF family from algae to modern angiosperms. We also found *GRF* genes in the genome of Charophyte algae species (*K. nitens)* and in the transcriptomes of other Charophytes *Spirogyra pratensis*, *Nitella mirabilis*, *Mesostigma viride*, *Closterium peracerosum-strigosum-littorale*, and *Klebsormidium crenulatum*, showing that the GRF family arose earlier than previously thought during the evolution of Streptophyta, most likely by a duplication event in the common ancestor of Charophyte and land plants. This finding and the phylogenetic results allowed us to suggest that *GRFs* may arise after the division between Charophyta and Chlorophyta due to the fact that the GRF is not present in genomes of Chlorophyta (Figures 1 and 9).

We found evidence for the emergence of GRFs in *Mesostigma viride*, from the basal Charophyte Mesostigmales (Figure 9). We also conducted searches on available public transcriptome databases and found GRF encoding sequences in the Charophytes *Spirogyra pratensis*, *Nitella mirabilis*, *Mesostigma viride*, *Closterium peracerosum-strigosum-littorale*, and *Klebsormidium crenulatum*. Previous studies proposed that the GRF genes have originated after the emergence of Embryophyta (Omidbakhshfard *et al.*, 2015; Kim and Tsukaya, 2015), mainly because of the absence of GRFs in Chlorophyta species. However, the availability of the Charophyta genome allowed to demonstrate that GRFs most likely originated earlier (Catarino *et al.*, 2016; Wilhelmsson *et al.*, 2017). Hence, this family existed even before the first multicellular green plants, which arose after the divergence between Mesostigmales and Chlorkybales (Jill Harrison, 2017).

Previous works reported the presence of QLQ in both SNF2 and GRF genes (van der Knaap *et al.*, 2000; Omidbakhshfard *et al.*, 2015; Cao *et al.*, 2016; Fina *et al.*, 2017; Khatun *et al.*, 2017), but, to date, no study had been dedicated to explore the divergence between these 2 families. GIFs are known to be molecular partners of GRFs in the regulation of cell proliferation (Horiguchi *et al.*, 2005), ear development (Zhang *et al.*, 2008), flower development (Liu *et al.*, 2014), and plant longevity (Debernardi *et al.*, 2014). Also, it is already known that the interaction of GRFs and GIFs occurs via QLQ and SNH domains, respectively. SNF2 proteins interact with SYT proteins, the GIF homologs. Because the region of the interaction of SNF2 with SYT was already described, we analyzed the protein sequences and found that the regions correspond to QLQ and SNH domains, respectively. Beyond that, AtGIF1 is shown to interact with SWI/SNF complexes through the interaction with BRM and SYD (Vercruyssen *et al.*, 2014). These dimer formations prompted us to investigate the divergence between GRFs and SNF2 genes.

We also demonstrated that the QLQ domain from GRF and SNF2 diversified particularly early in the course of evolution, although both maintained the protein interaction function with the SNH domains present in the homologous GIFs or SYT, respectively. Whereas SNF2 remained as chromatin remodeling proteins, GRFs evolved as specific transcription factors.

We hypothesized that the QLQ present in *GRFs* arose from a duplication of an *SNF2* QLQ in the common ancestor of the Charophytes and land plants, and the divergence between these genes appears to have occurred early in the evolution. Our phylogenetic analysis revealed that SNF2 and GRF genes are grouped into distinct clades with the presence of algae and moss sequences in both clades. This observation suggests that the divergence between both SNF2 and GRF QLQ domains occurred before the emergence of land plants and after the divergence between the Chlorophyta and Charophyta lineages. Interestingly, although sequences from *Spirogyra pratensis, Closterium peracerosum-strigosum-littorale*, and *Klebsormidium crenulatum* grouped within the GRF cluster, as well as the sequence derived from *Klebsormidium nitens*, GRF coding sequences from *Nitella mirabilis and Mesostigma viride* were kept out. A detailed analysis from these sequences revealed that QLQ positions 9 and 11 are not occupied by glutamic acids, as observed for almost every GRF encoding sequence analyzed (Figure 3). In fact, these positions are occupied either by an isoleucine or glutamine and by a glutamine or aspartate, respectively. Despite having a WRC domain and high similarity to other GRFs, both proteins harbor a QLQ that resembles SNF2 proteins, presenting at least one neutral residue within these positions. Also, our analyses from the rates of nonsynonymous to synonymous substitutions suggest a positive selection in QLQ from GRFs (Table S4).

The expansion of the GRF family accompanied the rapid evolution of plants, since the basal Charophytes until the modern angiosperms (Figures 1 and 9). Remarkably, GRFs evolved with the expansion of gene number and remained as families. Whereas in the Charophyte *K. nitens* genome there is just one gene, the family encompasses 24 genes in soybean, the one with the highest number of genes among the species analyzed. Other species with a high number of genes, such as switchgrass, maize, turnip, cotton, and Salicaceae, underwent whole-genome duplication (WGD) events at some moment in the course of evolution (Renny-Byfield and Wendel, 2014). This finding supports the data obtained by the phylogenetic analyses, suggesting that besides ancestral duplications in basal monocot and eudicot, recent WGD events were crucial for the expansion of the family.

The conservation among the sequences of QLQ and WRC in different GRFs did not allow further characterization of the relations between the groups. However, through the analysis of the domain composition, we observed that the dicot Group IV and the monocot Group V are somewhat related. We also noticed a relationship between monocot groups X and XI, both with no additional domains and a short C-terminal region. ZmGRF10, a member of Group X, can interact with GIFs. However, it lacks the C-terminal domain and the transactivation activity (Wu *et al.*, 2014). The other members of Groups X and XI share the same structure of ZmGRF10; hence, it is possible that the other GRFs in both groups also lack this transactivation ability. Our analyses also suggest that duplications on the basis of monocots and eudicots and species-specific WGD events were crucial for the expansion of the GRF family in Viridiplantae.

Functional studies on Arabidopsis and rice illustrated that GRFs play diverse roles in important agronomic traits such as plant growth, grain productivity, stress responses, and integration of defense with growth processes (see Kim and Tsukaya, 2015 and Omidbakhshfard *et al.*, 2015 for reviews). In this work, we identified several paralogous and probable orthologs of genes related to these traits that could be manipulated in order to favor characteristics of interest. In this work, we identified putative targets of this transcription factor family in rice and orthologs of GRFs known to play important agronomic roles, findings that may be important in guiding future studies in diverse species.

OsGRF4 and OsGRF6 have been linked to yield-related traits, regulating grain size (Che *et al.*, 2015; Duan *et al.*, 2015) and panicle branching (Gao *et al.*, 2015), respectively. The expression of both genes and their homologs could be explored to improve plant productivity, alone or in combination, mainly in cereal crops. We identified paralogous and ortholog versions of both genes. OsGRF3 is paralogous of OsGRF4, whereas BdGRF5 and 11, and ZmGRF1 and 5 are their putative orthologs. Also, OsGRF6 and OsGRF9 are contained in a syntenic block of duplication and seem to be paralogous, and the probable orthologs of OsGRF6 are BdGRF1, ZmGRF17, and ZmGRF18.

Regarding stress responses, AtGRF7 has been implicated in the regulation of osmotic stress-responsive genes to prevent growth inhibition under stress conditions (Kim *et al.*, 2012). We found probable orthologs of AtGRF7 in the genomes of tomato (SlGRF8) and soybean (GmGRF9 and 10). The expression of AtGRF7 or its orthologs, in combination with osmotic defense genes, could be utilized to balance growth and defense processes during stress. Alterations in the expression of AtGRF1 and 2 in response to infection with cyst nematodes were already related to the development of the s*yncytium*, a feeding structure that enables nematode establishment in roots (Hewezi *et al.*, 2012). Modulation of the expression of both genes, or their putative orthologs SlGRF5 and 6, could be important for preventing the formation of the feeding structure, avoiding nematode infection. All these genes are promising candidates for genetic engineering of important agronomic traits and could be further investigated in future studies.

## Data Access

The alignments are availabe at: https://data.mendeley.com/datasets/p25czj44sn/draft?a=801f6365-5d02-48f5-aef4-e6da1f3a510b.

## Supplementary Material

The following online material is available for this article:

Table S1 Species, loci and taxa terminologies of SNF2-type genes used in the tree.

Table S2 Species, loci and taxa terminologies of GRF genes used in the trees.

Table S3 GRF numbers and loci used in the syntenyc analysis.

Table S4 Site and branch-site model analysis.

Table S5 GO terms and enrichment analysis of putative targets of GRFs in rice.

Table S6 Annotation of 1270 putative targets of GRFs in rice genome.

Figure S1 Phylogenetic tree of GRFs and protein domain composition.

Figure S2 Phylogenetic relationship of GRFs.
