# Supplementary material for: Molecular evolution and diversification of the GRF transcription factor family
Source: Genet Mol Biol. 2020 Jul 24;43(3):20200080. doi: 10.1590/1678-4685-GMB-2020-0080 (PMC7380329; doi:10.1590/1678-4685-GMB-2020-0080)
Supplement: Supplementary file 1 [file 1415-4757-GMB-43-3-e20200080-suppl1.pdf]

Genetics and Molecular Biology
https://doi.org/10.1590/1678-4685-GMB-2020-0080

Supplementary Material to "Molecular evolution and diversification of the GRF transcription factor family"

Table S1 – Species, loci and taxa terminologies of SNF2-type genes used in the tree.

	**Organism**	**Genome version**	***Loci***	**Names used in the tree**
**Fungi**	*Allomyces macrogynus ATCC 38327*	GCA_000151295.1	AMAG12941; AMAG20005	Ama_12941; Ama_20005
	Aspergillus nidulans FGSC A4	ASM14920v1	XM_654790	Ani_XM654790
	Dichomitus squalens LYAD-421 SS1	GCA_000275845.1	XM_007364686; XM_007368367	Dsq_XM007364686; Dsq_XM007368367
	Eremothecium gossypii ATCC 10895	ASM9102v4	NM_212245	Ego_NM212245
	Pneumocystis carinii B80	GCA_001477545.1	XM_018369289	Pca_XM018369289
	Penicillium digitatum Pd1	GCA_000315645.2	XM_014678523	Pdi_XM014678523
	Saccharomyces cerevisiae S288C	GCA_000146045.2	NM_001183709	Sce_NM001183709
	Ustilago maydis 521	GCA_000328475.2	XM_011389107	Uma_XM011389107
	Cryptococcus gattii WM276	GCA_000185945.1	XM_0031965851	Cga_XM0031965851
**Algae**	*Chlamydomonas reinhardtii*	*v5.5*	CRE07.G325700	Cre_07G325700
	Micromonas sp. RCC299	*v3.0*	EUGENE.0200010520; EUGENE.0600010227	Msp_0200010520; Msp_0600010227
	Micromonas pusilla CCMP1545	*v3*	MICPUC2.EUGENE.0000090245	Mpu_0000090245
	Volvox carteri	*v2.1*	VOCAR.0021S0108	Vca_0021S0108
	Klebsormidium nitens	*v1.1*	kfl00148_0270	Kfl_001480270
**Land Plants**	*Arabidopsis thaliana*	*TAIR10*	AT2G28290 (SYD); AT2G46020 (BRM)	Ath_AT2G28290; Ath_AT2G46020
	Brachypodium distachyon	*v3.1*	BRADI1G44177; BRADI3G01297	Bdi_1G44177; Bdi_3G01297
	Brassica rapa	*FPsc v1.3*	BRARA.C02316; BRARA.D02781	Bra_C02316; Bra_D02781
	Oryza sativa	*v7_JGI*	LOC_Os02g02290	Osa_Os02g02290
	Physcomitrella patens	*v3.3*	Pp3c13_14440; Pp3c14_90; Pp3c3_15820	Ppa_3c1314440; Ppa_3c1490; Ppa_3c315820
	Populus trichocarpa	*v3.1*	POTRI.002G160900; POTRI.008G195900; POTRI.010G019400; POTRI.014G086500	Ptr_002G160900; Ptr_008G195900; Ptr_010G019400; Ptr_014G086500
	Sphagnum fallax	*v0.5*	SPHFALX0029S0063; SPHFALX0058S0094	Sfa_0029s0063; Sfa_0058s0094
**Metazoans**	*Caenorhabditis elegans*	*Metazome v3*	WBGENE00004204	Cel_00004204
	Canis familiaris	*Metazome v3*	LOC476335; LOC476710	Cfa_LOC476335; Cfa_LOC476710

Organism	Genome version	*Loci*	Names used in the tree
Danio rerio	*Metazome v3*	ENSDARG00000004314; ENSDARG00000008904; ENSDARG00000046105	Dre_00000004314; Dre_00000008904; Dre_00000046105
Gallus gallus	*Metazome v3*	ENSGALG00000010164	Gga_00000010164
Homo sapiens	*Metazome v3*	ENSG00000080503; ENSG00000127616	Hsa_00000080503; Hsa_00000127616
Monodelphis domestica	*Metazome v3*	ENSMODG00000003578; ENSMODG00000006287	Mdo_00000003578; Mdo_00000006287
Nematostella vectensis	*Metazome v3*	178940	Nve_178940
Rattus norvegicus	*Metazome v3*	ENSRNOG00000009271; ENSRNOG00000011931	Rno_00000009271; Rno_00000011931
Strongylocentrotus purpuratus	*Metazome v3*	LOC589355; LOC756605	Stp_LOC589355; Stp_LOC756605
Tribolium castaneum	*Metazome v3*	LOC655742	Trc_LOC655742
Xenopus tropicalis	*Metazome v3*	461216; 470898	Xtr_461216; Xtr_470898
