# Supplementary material for: Molecular evolution and diversification of the GRF transcription factor family
Source: Genet Mol Biol. 2020 Jul 24;43(3):20200080. doi: 10.1590/1678-4685-GMB-2020-0080 (PMC7380329; doi:10.1590/1678-4685-GMB-2020-0080)
Supplement: Supplementary file 2 [file 1415-4757-GMB-43-3-e20200080-suppl2.pdf]

Genetics and Molecular Biology

https://doi.org/10.1590/1678-4685-GMB-2020-0080

Supplementary Material to "Molecular evolution and diversification of the GRF transcription factor family"

Table S2 - Species, *loci* and taxa terminologies of GRF genes used in the trees

Organism and genome version	GRFs	*Loci*	Names used on trees
Aquilegia coerulea v3.1	7	Acqoe1G454000 ; Acqoe2G129900; Acqoe3G214400; Acqoe5G457000 ; Acqoe6G259800; Acqoe7G060800; Acqoe7G112900	Aco_1G454000; Aco_2G129900; Aco_3G214400; Aco_5G457000; Aco_6G259800; Aco_7G060800; Aco_7G112900
Arabidopsis lyrata v2.1	9	AL3G26150; AL3G43750; AL4G12420; AL4G32560; AL4G44650; AL5G33700; AL7G12940; AL7G29140; AL8G28610	Aly_3G26150; Aly_3G43750; Aly_4G12420; Aly_4G32560; Aly_4G44650; Aly_5G33700; Aly_7G12940; Aly_7G29140; Aly_8G28610
Arabidopsis thaliana TAIR10	9	AT2G06200; AT2G22840; AT2G36400; AT2G45480; AT3G13960; AT3G52910; AT4G24150; AT4G37740; AT5G53660	Ath_AT2G06200; Ath_AT2G22840; Ath_AT2G36400; Ath_AT2G45480; Ath_AT3G13960; Ath_AT3G52910; Ath_AT4G24150; Ath_AT4G37740; Ath_AT5G53660
Azolla Filiculoides v1.1	3	Azfi_s0256.g060519; Azfi_s0001.g000094; Azfi_s0097.g043820†	Azf_s0256g060519; Azf_s0001g000094
Boechera stricta v1.2	8	Bostr.23794s0452; Bostr.24399s0028; Bostr.25993s0227; Bostr.26833s0909; Bostr.28625s0309; Bostr.7867s0220; Bostr.7867s1635; Bostr.19427s0054	Bos_23794s0452; Bos_24399s0028; Bos_25993s0227; Bos_26833s0909; Bos_28625s0309; Bos_7867s0220; Bos_7867s1635; Bos_9427s0054
Brachypodium distachyon v3.1	12	Bradi1g09900; Bradi1g12650; Bradi1g28400; Bradi1g46427; Bradi1g50597; Bradi2g14320; Bradi3g51685; Bradi3g52547; Bradi3g57267; Bradi4g16450; Bradi5g18961; Bradi5g20607	Bdi_1g09900; Bdi_1g12650; Bdi_1g28400; Bdi_1g46427; Bdi_1g50597; Bdi_2g14320; Bdi_3g51685; Bdi_3g52547; Bdi_3g57267; Bdi_4g16450; Bdi_5g18961 ; Bdi_5g20607
Brachypodium stacei v1.1	12	Brast02G260100; Brast02G292700; Brast04G093600; Brast04G039200; Brast04G104800; Brast06G139500; Brast07G020400; Brast07G067500; Brast08G002800; Brast09G178600; Brast09G195300; Brast10G125600	Bst_02G260100; Bst_02G292700; Bst_04G0936000; Bst_04G039200; Bst_04G104800; Bst_06G139500; Bst_07G020400; Bst_07G067500; Bst_08G002800; Bst_09G178600; Bst_09G195300; Bst_10G125600
Brassica rapa FPsc v1.3	16	Brara.A00122; Brara.A01418; Brara.A03222; Brara.B01438; Brara.C01828; Brara.C02492; Brara.C03502; Brara.D02218; Brara.D02744; Brara.E00841; Brara.E02725; Brara.G00464; Brara.G01562; Brara.I03590; Brara.K00742; Brara.K01237	Bra_A00122; Bra_A01418; Bra_A03222; Bra_B01438; Bra_C01828; Bra_C02492; Bra_C03502; Bra_D02218; Bra_D02744; Bra_E00841; Bra_E02725; Bra_G00464; Bra_G01562; Bra_I03590; Bra_K00742; Bra_K01237
Capsella grandiflora v1.1	8	Cagra.0228s0030; Cagra.0612s0011; Cagra.0736s0034; Cagra.0757s0005; Cagra.2460s0032; Cagra.4290s0006; Cagra.9757s0010; Cagra.1226s0080	Cgr_0228s0030; Cgr_0612s0011; Cgr_0736s0034; Cgr_0757s0005; Cgr_2460s0032; Cgr_4290s0006; Cgr_9757s0010; Cgr_1226s0080
Capsella rubella v1.0	8	Carubv10004544m.g; Carubv10007298m.g; Carubv10013860m.g; Carubv10015887m.g; Carubv10017366m.g; Carubv10022975m.g; Carubv10025172m.g; Carubv10026610m.g	Cru_10004544; Cru_10007298; Cru_10013860; Cru_10015887; Cru_10017366; Cru_10022975; Cru_10025172; Cru_10026610
Citrus clementina v1.0	9	Ciclev10001613m.g; Ciclev10008922m.g; Ciclev10011856m.g; Ciclev10019529m.g; Ciclev10019828m.g; Ciclev10022033m.g; Ciclev10025209m.g; Ciclev10025855m.g; Ciclev10032065m.g Acqoe7G060800	Ccl_10001613; Ccl_10008922; Ccl_10011856; Ccl_10019529; Ccl_10019828; Ccl_10022033; Ccl_10025209; Ccl_10025855; Ccl_10032065Aco_7G060800
Citrus sinensis v1.1	9	orange1.1g007514m.g; orange1.1g009918m.g; orange1.1g010979m.g; orange1.1g018208m.g; orange1.1g020051m.g; orange1.1g023105m.g; orange1.1g028751m.g; orange1.1g047108m.g; orange1.1g040599m.g†	Csi_g007514m; Csi_g009918m; Csi_g010979m; Csi_g018208m; Csi_g020051m;

Organism and genome version	GRFs	*Loci*	Names used on trees
			Csi_g023105m; Csi_g028751m; Csi_g047108m;
Closterium peracerosum-strigosum-littorale (ESTs)	1	n.a.	Clo_01
Cucumis sativus v1.0	8	Cucsa.017410; Cucsa.257800; Cucsa.378420; Cucsa.395430; Cucsa.103390; Cucsa.117300; Cucsa.141640; Cucsa.192190	Csa_017410; Csa_257800; Csa_378420; Csa_395430; Csa_103390; Csa_117300; Csa_141640; Csa_192190
Eucalyptus grandis v2.0	5	Eucgr.A01418; Eucgr.A01534; Eucgr.C00823; Eucgr.F00097; Eucgr.F04420	Egr_A01418; Egr_A01534; Egr_C00823; Egr_F00097; Egr_F04420
Eutrema salsugineum v1.0	9	Thhalv10000124m.g; Thhalv10001460m.g; Thhalv10002643m.g; Thhalv10010453m.g; Thhalv10013862m.g; Thhalv10016778m.g; Thhalv10020873m.g; Thhalv10024915m.g; Thhalv10025371m.g	Esa_10000124; Esa_10001460; Esa_10002643; Esa_10010453; Esa_10013862; Esa_10016778; Esa_10020873; Esa_10024915; Esa_10025371
Fragaria vesca v1.1	10	gene01886v1.0hybrid; gene02620v1.0hybrid; gene05636v1.0hybrid; gene07429v1.0hybrid; gene12474v1.0hybrid; gene12549v1.0hybrid; gene12741v1.0hybrid; gene13517v1.0hybrid; gene19384v1.0hybrid; gene30232v1.0hybrid	Fve_01886; Fve_02620; Fve_05636; Fve_07429; Fve_12474; Fve_12549; Fve_12741; Fve_13517; Fve_19384; Fve_30232
Glycine max Wm82.a2.v1	24	Glyma.01G148600; Glyma.01G234400; Glyma.03G192200; Glyma.04G230600; Glyma.06G134600; Glyma.07G038400; Glyma.09G068700; Glyma.09G212500; Glyma.U028600; Glyma.U028700; Glyma.10G067200; Glyma.11G008500; Glyma.11G110700; Glyma.11G208800; Glyma.12G014700; Glyma.13G109500; Glyma.15G176500; Glyma.16G007600; Glyma.17G050200; Glyma.17G232600; Glyma.17G232700; Glyma.03G021900; Glyma.01G144900; Glyma.19G192700	Gma_01G148600; Gma_01G234400; Gma_03G192200; Gma_04G230600; Gma_06G134600; Gma_07G038400; Gma_09G068700; Gma_09G212500; Gma_U028600; Gma_U028700; Gma_10G067200; Gma_11G008500; Gma_11G110700; Gma_11G208800; Gma_12G014700; Gma_13G109500; Gma_15G176500; Gma_16G007600; Gma_17G050200; Gma_17G232600; Gma_17G232700; Gma_03G021900; Gma_01G144900; Gma_19G192700
Gossypium raimondii v2.1	18	Gorai.003G032300; Gorai.003G057700; Gorai.004G204600; Gorai.005G098700; Gorai.006G157300; Gorai.007G092400; Gorai.008G241800; Gorai.008G259500; Gorai.008G265600; Gorai.009G035200; Gorai.009G222200; Gorai.009G277500; Gorai.009G406500; Gorai.010G027700; Gorai.011G058800; Gorai.011G108400; Gorai.013G184200; Gorai.013G225300	Gra_003G032300; Gra_003G057700; Gra_004G204600; Gra_005G098700; Gra_006G157300; Gra_007G092400; Gra_008G241800; Gra_008G259500; Gra_008G265600; Gra_009G035200; Gra_009G222200; Gra_009G277500; Gra_009G406500; Gra_010G027700; Gra_011G058800; Gra_011G108400; Gra_013G184200; Gra_013G225300
Klebsormidium crenulatum (ESTs)	1	n.a.	Klc_01
Klebsormidium nitens v1.1	1	kfl00186_0090	Kfl_001860090
Manihot esculenta v6.1	15	Manes.01G041800; Manes.01G070800; Manes.01G264700; Manes.02G031200; Manes.03G039500; Manes.04G144700; Manes.05G043700; Manes.05G183900; Manes.08G022300; Manes.08G160800; Manes.09G059500; Manes.11G020200; Manes.12G117600; Manes.16G096400; Manes.18G049600	Mes_01G041800; Mes_01G070800; Mes_01G264700; Mes_02G031200; Mes_03G039500; Mes_04G144700; Mes_05G043700; Mes_05G183900; Mes_08G022300; Mes_08G160800; Mes_09G059500; Mes_11G020200; Mes_12G117600; Mes_16G096400; Mes_18G049600
Medicago truncatula Mt4.0v1	9	Medtr0001s0490; Medtr1g017490; Medtr2g041430; Medtr3g092330; Medtr4g125490; Medtr5g027030; Medtr7g104050; Medtr8g020560; Medtr8g035910	Mtr_0001s0490; Mtr_1g017490; Mtr_2g041430; Mtr_3g092330; Mtr_4g125490; Mtr_5g027030; Mtr_7g104050; Mtr_8g020560; Mtr_8g035910
Mesostigma viride (ESTs)	1	n.a.	Mes_01
Nitella mirabilis (ESTs)	1	n.a.	Nit_01
Oryza sativa v7_JGI	12	LOC_Os02g45570; LOC_Os02g47280; LOC_Os02g53690; LOC_Os03g47140; LOC_Os03g51970; LOC_Os04g48510;	Osa_Os02g45570; Osa_Os02g47280; Osa_Os02g53690; Osa_Os03g47140; Osa_Os03g51970; Osa_Os04g48510 ;

Organism and genome version	GRFs	*Loci*	Names used on trees
		LOC_Os04g51190; LOC_Os06g02560; LOC_Os06g10310; LOC_Os07g28430; LOC_Os11g35030; LOC_Os12g29980	Osa_Os04g51190; Osa_Os06g02560 ; Osa_Os06g10310; Osa_Os07g28430; Osa_Os11g35030; Osa_Os12g29980
Panicum hallii v2.0	10	Pahal.A02912; Pahal.A03059; Pahal.A03557; Pahal.B03870; Pahal.D02874; Pahal.G02182; Pahal.H01224; Pahal.H02316; Pahal.I01892; Pahal.I02265	Pha_A02912; Pha_A03059; Pha_A03557; Pha_B03870; Pha_D02874; Pha_G02182; Pha_H01224; Pha_H02316; Pha_I01892; Pha_I02265
Panicum virgatum v1.1	19	Pavir.Aa00789; Pavir.Aa00919; Pavir.Ab02786; Pavir.Ba01449; Pavir.Bb02610; Pavir.Da01847; Pavir.Da02366; Pavir.Db02048†; Pavir.Db02421; Pavir.Ga00724; Pavir.Gb00674; Pavir.Ha01401; Pavir.Hb00712; Pavir.Ia01043; Pavir.J03212; Pavir.J06517; Pavir.J11763; Pavir.J14128; Pavir.J29705	Pvi_Aa00789; Pvi_Aa00919; Pvi_Ab02786; Pvi_Ba01449; Pvi_Bb02610; Pvi_Da01847; Pvi_Da02366; Pvi_Db02421; Pvi_Ga00724; Pvi_Gb00674; Pvi_Ha01401; Pvi_Hb00712; Pvi_Ia01043; Pvi_J03212; Pvi_J06517; Pvi_J11763; Pvi_J14128; Pvi_J29705
Phaseolus vulgaris v2.1	11	Phvul.001G031000; Phvul.001G187500; Phvul.002G041800; Phvul.002G131700; Phvul.003G131800; Phvul.007G222300; Phvul.009G047000; Phvul.009G228000; Phvul.010G048400; Phvul.010G130000; Phvul.011G017700	Pvu_001G031000; Pvu_001G187500; Pvu_002G041800; Pvu_002G131700; Pvu_003G131800; Pvu_007G222300; Pvu_009G047000; Pvu_009G228000; Pvu_010G048400; Pvu_010G130000; Pvu_011G017700
Physcomitrella patens v3.3	2	Pp3c12_3720; Pp3c17_1780	Ppa_3c123720; Ppa_3c171780
Picea abies v1.0	5	MA_33661g0010; MA_18836g0010; MA_88101g0010; MA_111469g0010; MA_137877p0010	Pic_33661g0010; Pic_18836g0010; Pic_88101g0010; Pic_111469g0010; Pic_137877p0010
Pinus taeda v1.0	5	PITA_000074544; PITA_000023549; PITA_000090725; PITA_000014799; PITA_000034605	Pin_000074544; Pin_000023549; Pin_000090725; Pin_000014799; Pin_000034605
Populus trichocarpa v3.1	19	Potri.001G082700; Potri.001G114000; Potri.001G132600; Potri.001G169100; Potri.002G115100; Potri.003G065000; Potri.003G100800; Potri.003G118100; Potri.006G115200; Potri.006G143200; Potri.007G007100; Potri.012G022600; Potri.013G077500; Potri.014G007200; Potri.014G012800; Potri.014G071800; Potri.015G006200; Potri.018G065400; Potri.019G042300	Ptr_001G082700; Ptr_001G114000; Ptr_001G132600; Ptr_001G169100; Ptr_002G115100; Ptr_003G065000; Ptr_003G100800; Ptr_003G118100; Ptr_006G115200; Ptr_006G143200; Ptr_007G007100; Ptr_012G022600; Ptr_013G077500; Ptr_014G007200; Ptr_014G012800; Ptr_014G071800; Ptr_015G006200; Ptr_018G065400; Ptr_019G042300
Prunus persica v2.1	10	Prupe.2G148100; Prupe.2G317500; Prupe.2G329200; Prupe.3G041800; Prupe.5G021400; Prupe.5G131600; Prupe.6G217000; Prupe.7G115500; Prupe.7G201000; Prupe.1G419000	Ppe_2G148100; Ppe_2G317500; Ppe_2G329200; Ppe_3G041800; Ppe_5G021400; Ppe_5G131600; Ppe_6G217000; Ppe_7G115500; Ppe_7G201000; Ppe_1G419000
Ricinus communis v0.1	9	27496.t000001; 28976.t000005; 29700.t000017; 29706.t000013; 29739.t000211; 30068.t000078; 30170.t000085; 30174.t000307; 30190.t000302	Rco_27496m000094; Rco_28976m000158; Rco_29700m000761; Rco_29706m001283; Rco_29739m003775; Rco_30068m002588; Rco_30170m013673; Rco_30174m008910; Rco_30190m011066
Salix purpurea v1.0	21	SapurV1A.0002s0920; SapurV1A.0004s0240; SapurV1A.0045s0430; SapurV1A.0051s0520; SapurV1A.0059s0450; SapurV1A.0063s0420; SapurV1A.0087s0080; SapurV1A.0094s0370; SapurV1A.0177s0310; SapurV1A.0208s0190; SapurV1A.0235s0230; SapurV1A.0260s0250; SapurV1A.0326s0130; SapurV1A.0532s0070; SapurV1A.0568s0060; SapurV1A.0608s0150; SapurV1A.0611s0190; SapurV1A.0616s0110; SapurV1A.0755s0030; SapurV1A.3102s0020; SapurV1A.3612s0010	Spu_0002s0920; Spu_0004s0240; Spu_0045s0430; Spu_0051s0520; Spu_0059s0450; Spu_0063s0420; Spu_0087s0080; Spu_0094s0370; Spu_0177s0310; Spu_0208s0190; Spu_0235s0230; Spu_0260s0250; Spu_0326s0130; Spu_0532s0070; Spu_0568s0060; Spu_0608s0150; Spu_0611s0190; Spu_0616s0110; Spu_0755s0030; Spu_3102s0020; Spu_3612s0010
Salvinia Cucullata v1.1	4	Sacu_v1.1_s0120.g021398; Sacu_v1.1_s0001.g000754; Sacu_v1.1_s0161.g023900;	Sal_s0120g021398; Sal_s0001g000754; Sal_s0161g023900

Organism and genome version	GRFs	*Loci*	Names used on trees
		Sacu_v1.1_s0004.g002301 †	
Selaginella moellendorffii v1.0	4	412762; 417311; 420739; 442384	Smo_412762; Smo_417311; Smo_420739; Smo_442384
Setaria italica v2.2	10	Seita.2G309100; Seita.4G012400; Seita.4G086400; Seita.7G224500; Seita.8G149500; Seita.9G107700; Seita.9G141900; Seita.1G271800; Seita.1G287100; Seita.1G338700	Sit_2G309100; Sit_4G012400; Sit_4G086400; Sit_7G224500; Sit_8G149500; Sit_9G107700; Sit_9G141900; Sit_1G271800; Sit_1G287100; Sit_1G338700
Solanum lycopersicum iTAG2.4	13	Solyc01g091540.1; Solyc02g092070.2; Solyc03g082430.1; Solyc04g077510.2; Solyc07g041640.2; Solyc08g005430.2; Solyc08g068760.1; Solyc08g075950.1; Solyc08g079800.2; Solyc08g083230.1; Solyc09g009200.1; Solyc10g083510.1; Solyc12g096070.1	Sly_01g091540; Sly_02g092070; Sly_03g082430; Sly_04g077510; Sly_07g041640; Sly_08g005430; Sly_08g068760; Sly_08g075950; Sly_08g079800 ; Sly_08g083230; Sly_09g009200; Sly_10g083510; Sly_12g096070
Solanum tuberosum v4.3	9	PGSC0003DMG400003220; PGSC0003DMG400004942; PGSC0003DMG400012233; PGSC0003DMG400012767; PGSC0003DMG400018491; PGSC0003DMG400021403; PGSC0003DMG400028259; PGSC0003DMG400029384; PGSC0003DMG400030881	Stu_400005813; Stu_400008794; Stu_400021643; Stu_400022619; Stu_400032217; Stu_400037092; Stu_400049127; Stu_400051174; Stu_400053791
Sphagnum fallax v0.5	4	Sphfalx0018s0181; Sphfalx0019s0149; Sphfalx0126s0017; Sphfalx0221s0016	Sfa_0018s0181; Sfa_0019s0149; Sfa_0126s0017; Sfa_0221s0016
Spirogira pratensis (ESTs)	1	n.a.	Spi_01
Theobroma cacao v1.1	10	Thecc1EG000838; Thecc1EG005483; Thecc1EG015497; Thecc1EG015625; Thecc1EG016120; Thecc1EG022490; Thecc1EG025561; Thecc1EG029130; Thecc1EG034655; Thecc1EG037550	Tca_000838; Tca_005483; Tca_015497; Tca_015625; Tca_016120; Tca_022490; Tca_025561; Tca_029130; Tca_034655; Tca_037550
Vitis vinifera Genoscope.12X	10	GSVIVG01007165001; GSVIVG01009299001; GSVIVG01015095001; GSVIVG01016762001; GSVIVG01019913001; GSVIVG01024326001; GSVIVG01033800001; GSVIVG01038629001; GSVIVG01019667001; GSVIVG01027535001	Vvi_01007165001; Vvi_01009299001; Vvi_01015095001; Vvi_01016762001; Vvi_01019913001; Vvi_01024326001; Vvi_01033800001; Vvi_01038629001; Vvi_01019667001 (VviGRF9)*; Vvi_01027535001(VviGRF10)*
Zea mays Ensembl-18	16	GRMZM2G004619; GRMZM2G018414; GRMZM2G034876; GRMZM2G041223; GRMZM2G045977; GRMZM2G067743; GRMZM2G096709; GRMZM2G098594; GRMZM2G099862; GRMZM2G105335; GRMZM2G124566; GRMZM2G129147; GRMZM2G178261; GRMZM5G850129; GRMZM5G853392; GRMZM5G893117†	Zma_2G004619; Zma_2G018414; Zma_2G034876; Zma_2G041223; Zma_2G045977; Zma_2G067743; ma_2G096709; Zma_2G098594; Zma_2G099862; Zma_2G105335; Zma_2G124566; Zma_2G129147; Zma_2G178261; Zma_5G850129; Zma_5G853392

† Genes not used in the trees due to the absence of QLQ or WRC domais.

*Genes named in this work.
