# Supplementary material for: Molecular evolution and diversification of the GRF transcription factor family
Source: Genet Mol Biol. 2020 Jul 24;43(3):20200080. doi: 10.1590/1678-4685-GMB-2020-0080 (PMC7380329; doi:10.1590/1678-4685-GMB-2020-0080)
Supplement: Supplementary file 3 [file 1415-4757-GMB-43-3-e20200080-suppl3.pdf]

Genetics and Molecular Biology

https://doi.org/10.1590/1678-4685-GMB-2020-0080

Supplementary Material to "Molecular evolution and diversification of the GRF transcription factor family"

Table S3 - GRF numbers and *loci* used in the syntenyc analysis

**GRF nº**	***Glycine max*[a]**	***Zea mays*[*,b]**	***Solanum lycopersicum*[c]**	***Brachypodium distachyon*[*,d]**	***Oryza sativa*[e]**	***Arabidopsis thaliana*[f]**
**GRF1**	Glyma.15G176500	GRMZM2G034876	Solyc12g096070	Bradi1g09900	LOC_Os02g53690	AT2G22840
**GRF2**	Glyma.09G068700	GRMZM2G099862	Solyc08g005430	Bradi1g12650	LOC_Os06g10310	AT4G37740
**GRF3**	Glyma.13G109500	GRMZM2G105335	Solyc08g075950	Bradi2g14320	LOC_Os04g51190	AT2G36400
**GRF4**	Glyma.17G050200	GRMZM2G004619	Solyc07g041640	Bradi4g16450	LOC_Os02g47280	AT3G52910
**GRF5**	Glyma.17G232700	GRMZM2G129147	Solyc04g077510	Bradi5g20607	LOC_Os06g02560	AT3G13960
**GRF6**	Glyma.U028700	GRMZM2G041223	Solyc02g092070	Bradi1g28400	LOC_Os03g51970	AT2G06200
**GRF7**	Glyma.17G232600	GRMZM5G850129	Solyc08g083230	Bradi1g46427	LOC_Os12g29980	AT5G53660
**GRF8**	Glyma.U028600	GRMZM2G033612†	Solyc03g082430	Bradi1g50597	LOC_Os11g35030	AT4G24150
**GRF9**	Glyma.04G230600	GRMZM5G893117†	Solyc08g068760	Bradi3g51685	LOC_Os03g47140	AT2G45480
**GRF10**	Glyma.06G134600	GRMZM2G096709	Solyc01g091540	Bradi3g57267	LOC_Os02g45570	
**GRF11**	Glyma.16G007600	GRMZM2G067743	Solyc09g009200	Bradi3g52547*	LOC_Os07g28430	
**GRF12**	Glyma.07G038400	GRMZM2G119359†	Solyc10g083510	Bradi5g18961*	LOC_Os04g48510	
**GRF13**	Glyma.11G110700	GRMZM2G018414	Solyc08g079800			
**GRF14**	Glyma.12G014700	GRMZM2G098594				
**GRF15**	Glyma.01G234400	GRMZM2G045977*				
**GRF16**	Glyma.11G008500	GRMZM2G124566*				
**GRF17**	Glyma.09G212500	GRMZM2G178261*				

GRF nº	*Glycine max*[a]	*Zea mays*[*,b]	*Solanum lycopersicum*[c]	*Brachypodium distachyon*[*,d]	*Oryza sativa*[e]	*Arabidopsis thaliana*[f]
**GRF18**	Glyma.01G148600	GRMZM5G853392*				
**GRF19**	Glyma.03G192200					
**GRF20**	Glyma.19G192700					
**GRF21**	Glyma.10G067200					
**GRF22**	Glyma.11G208800					
**GRF23**	Glyma.01G144900					
**GRF24**	Glyma.03G021900					

† ZmGRF8: QLQ was not found

† ZmGRF9: WRC was not found

†ZmGRF12: QLQ and WRC were not found

[*] Genes named in this work

[a] Liu et al., 2017

[b] Zhang et al., 2008

[c] Khatun et al., 2017

[d] Filiz et al., 2014

[e] Kim et al., 2004

f Kim et al., 2003
