# Supplementary material for: Molecular evolution and diversification of the GRF transcription factor family
Source: Genet Mol Biol. 2020 Jul 24;43(3):20200080. doi: 10.1590/1678-4685-GMB-2020-0080 (PMC7380329; doi:10.1590/1678-4685-GMB-2020-0080)
Supplement: Supplementary file 4 [file 1415-4757-GMB-43-3-e20200080-suppl7.pdf]

Genetics and Molecular Biology

https://doi.org/10.1590/1678-4685-GMB-2020-0080

Supplementary Material to “Molecular evolution and diversification of the GRF transcription factor family"

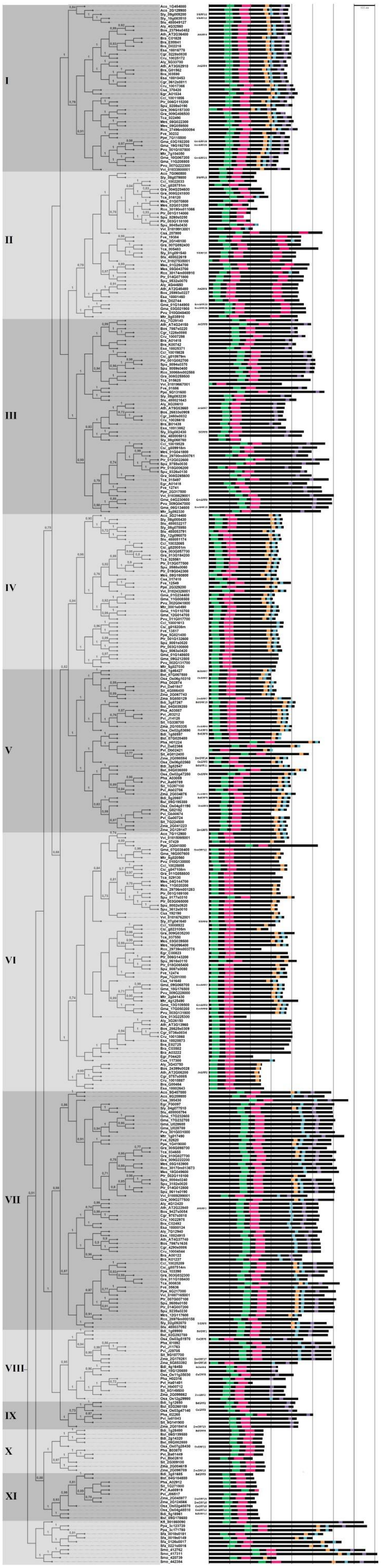

Figure S1 – Phylogenetic tree of GRFs and protein domain composition. The phylogenetic tree was reconstructed by Bayesian inference. The different domains in the protein diagram are colored in green (QLQ), pink (WRC), orange (TQL), blue (TQL), and purple (GGPL).
