# Supplementary material for: Molecular evolution and diversification of the GRF transcription factor family
Source: Genet Mol Biol. 2020 Jul 24;43(3):20200080. doi: 10.1590/1678-4685-GMB-2020-0080 (PMC7380329; doi:10.1590/1678-4685-GMB-2020-0080)
Supplement: Supplementary file 5 [file 1415-4757-GMB-43-3-e20200080-suppl4.pdf]

Genetics and Molecular Biology
https://doi.org/10.1590/1678-4685-GMB-2020-0080

Supplementary Material to "Molecular evolution and diversification of the GRF transcription factor family"

Table S4 – Site and branch-site model analysis

Gene family	Comparison	Parameters/significance
Site model analysis		
SNF2-GRF	M3 vs. M0	2ΔL=382 (df=4)***
	M2 vs. M1	2ΔL=0 (df=2)
	M8 vs. M7	2ΔL=0 (df=2)
Branch-site model analysis		
GRF (foreground), SNF2 (background)	Alternative vs. Null	2ΔL=5,59 (df=1)*

***p<0.001; * p< 0.05
