# Supplementary material for: Molecular evolution and diversification of the GRF transcription factor family
Source: Genet Mol Biol. 2020 Jul 24;43(3):20200080. doi: 10.1590/1678-4685-GMB-2020-0080 (PMC7380329; doi:10.1590/1678-4685-GMB-2020-0080)
Supplement: Supplementary file 6 [file 1415-4757-GMB-43-3-e20200080-suppl8.pdf]

*Genetics and Molecular Biology*
https://doi.org/10.1590/1678-4685-GMB-2020-0080

# Supplementary Material to “Molecular evolution and diversification of the GRF transcription factor family"

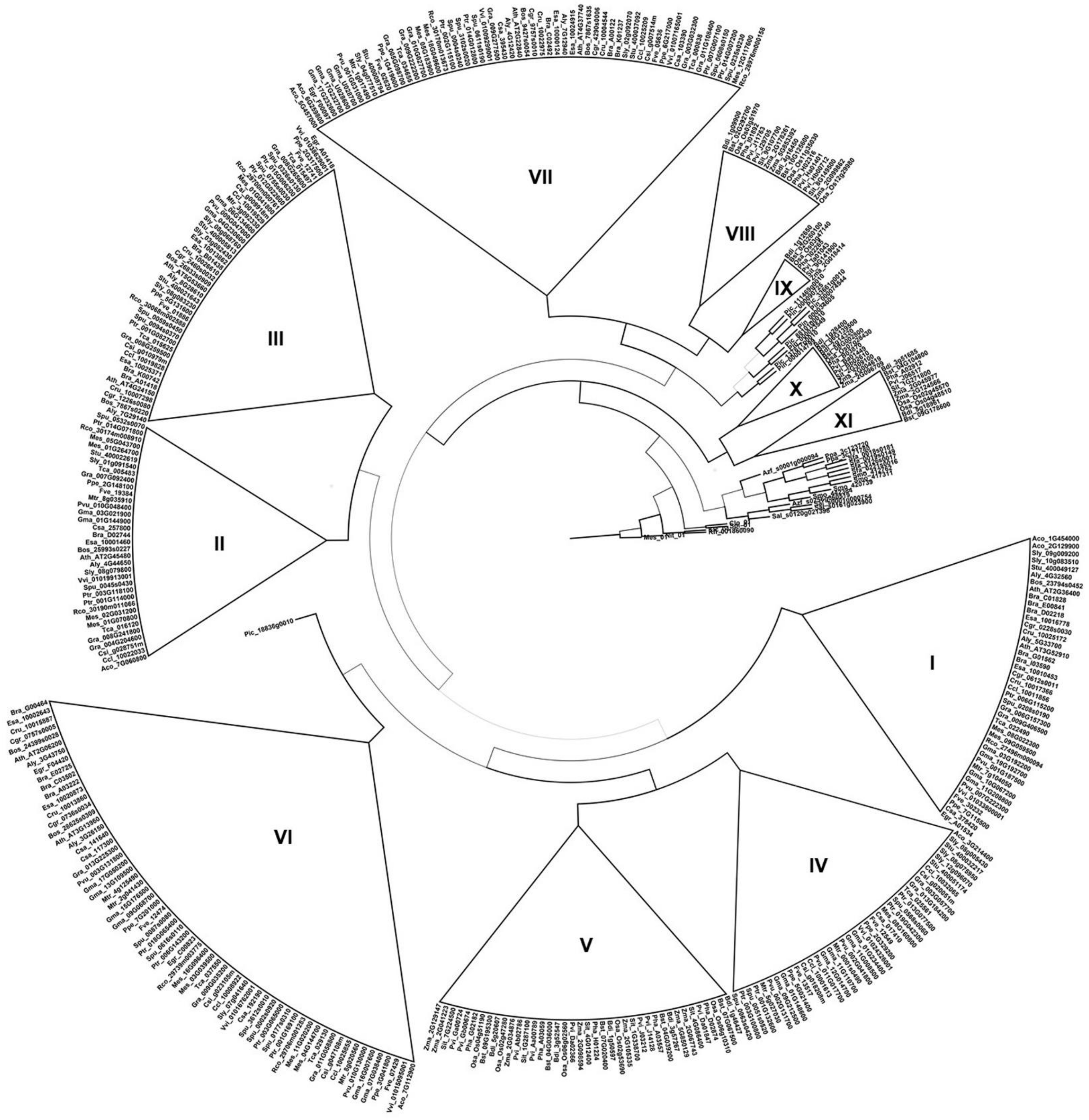


**Figure S2** – Phylogenetic relationship of GRFs. Bayesian phylogenetic tree showing relationship between GRFs based on QLQ and WRC domains. Coding sequences from Charophytes, ferns, Gymnosperms, and Angiosperms were used to generate an unrooted tree. Eleven groups supported by posterior probabilities above 0.9 are indicated. Species, loci, and taxa terminologies are available in Table S2. Posterior probabilities are illustrated by line width.
