# Supplementary material for: Molecular evolution and diversification of the GRF transcription factor family
Source: Genet Mol Biol. 2020 Jul 24;43(3):20200080. doi: 10.1590/1678-4685-GMB-2020-0080 (PMC7380329; doi:10.1590/1678-4685-GMB-2020-0080)
Supplement: Supplementary file 7 [file 1415-4757-GMB-43-3-e20200080-suppl5.pdf]

Genetics and Molecular Biology
https://doi.org/10.1590/1678-4685-GMB-2020-0080

Supplementary Material to "Molecular evolution and diversification of the GRF transcription factor family"

Table S5 – GO terms and enrichment analysis of putative targets of GRFs in rice

Gene Ontology biological process	Fold Enrichment	+/-	P value
response to gamma radiation	34.29	+	2.87E-02
response to stimulus	1.32	+	3.36E-03
regulation of leaf development	34.29	+	2.87E-02
regulation of endosperm development	34.29	+	2.87E-02
regulation of adaxial/abaxial pattern formation	34.29	+	2.87E-02
glycolipid translocation	34.29	+	2.87E-02
single-organism process	1.25	+	1.67E-04
cellular component organization	1.30	+	2.58E-02
cellular process	1.17	+	2.83E-04
cellular component organization or biogenesis	1.28	+	2.50E-02
single-organism cellular process	1.29	+	2.52E-03
glycolipid transport	34.29	+	2.87E-02
triglyceride catabolic process	34.29	+	2.87E-02
cellular lipid metabolic process	1.58	+	2.59E-02
lipid metabolic process	1.58	+	7.93E-03
organic substance metabolic process	1.23	+	2.51E-05
metabolic process	1.21	+	7.78E-06
primary metabolic process	1.22	+	6.87E-05
single-organism metabolic process	1.37	+	1.18E-04
cellular metabolic process	1.23	+	6.46E-05
acylglycerol catabolic process	34.29	+	2.87E-02
neutral lipid catabolic process	34.29	+	2.87E-02
cellular lipid catabolic process	5.28	+	1.14E-03
lipid catabolic process	2.64	+	1.88E-02
organic substance catabolic process	1.44	+	5.56E-03
catabolic process	1.44	+	2.40E-03
cellular catabolic process	1.39	+	1.71E-02
glycerolipid catabolic process	17.15	+	6.29E-03
pollen tube guidance	34.29	+	2.87E-02
reproductive process	1.65	+	4.32E-02
reproduction	1.65	+	4.32E-02
positive chemotaxis	34.29	+	2.87E-02
chemotaxis	34.29	+	2.87E-02
taxis	34.29	+	2.87E-02
locomotion	11.43	+	1.36E-02
developmental process involved in reproduction	2.47	+	1.24E-02
anatomical structure development	1.60	+	3.72E-02
protein-FAD linkage	34.29	+	2.87E-02
cellular protein modification process	1.24	+	1.87E-02
protein modification process	1.24	+	1.87E-02
protein metabolic process	1.27	+	1.70E-03
macromolecule metabolic process	1.23	+	2.90E-04
organonitrogen compound metabolic process	1.25	+	9.26E-04
nitrogen compound metabolic process	1.19	+	1.02E-03
macromolecule modification	1.18	+	3.91E-02
cellular protein metabolic process	1.20	+	2.40E-02
cellular macromolecule metabolic process	1.19	+	4.19E-03
7-methylguanosine cap hypermethylation	34.29	+	2.87E-02
RNA capping	9.80	+	1.82E-02
cell projection morphogenesis	34.29	+	2.87E-02
cell part morphogenesis	34.29	+	2.87E-02
phytochromobilin biosynthetic process	34.29	+	2.87E-02

Gene Ontology biological process	Fold Enrichment	+/-	P value
phytochromobilin metabolic process	34.29	+	2.87E-02
cofactor metabolic process	2.00	+	6.54E-03
cofactor biosynthetic process	2.08	+	2.53E-02
self proteolysis	34.29	+	2.87E-02
proteolysis	1.50	+	2.58E-03
Rho protein signal transduction	34.29	+	2.87E-02
cellular response to stimulus	1.27	+	3.52E-02
cyanate metabolic process	34.29	+	2.87E-02
organic acid metabolic process	1.55	+	9.31E-03
small molecule metabolic process	1.42	+	1.03E-02
mitochondrial respiratory chain complex II assembly	34.29	+	2.87E-02
respiratory chain complex II assembly	34.29	+	2.87E-02
mitochondrial respiratory chain complex II biogenesis	34.29	+	2.87E-02
regulation of double-strand break repair via homologous recombination	34.29	+	2.87E-02
regulation of DNA recombination	34.29	+	2.87E-02
regulation of nucleobase-containing compound metabolic process	0.76	-	3.38E-02
meiotic telophase I	34.29	+	2.87E-02
telophase	34.29	+	2.87E-02
regulation of meristem structural organization	34.29	+	2.87E-02
plant epidermal cell fate specification	34.29	+	2.87E-02
megasporogenesis	22.86	+	3.61E-03
embryo sac development	13.72	+	9.64E-03
gametophyte development	5.28	+	7.54E-03
plant-type sporogenesis	13.72	+	9.64E-03
meiotic cell cycle process	3.24	+	2.07E-02
meiotic cell cycle	3.17	+	1.30E-02
cell cycle	2.13	+	4.62E-03
cell cycle process	2.36	+	5.98E-03
sexual sporulation	13.72	+	9.64E-03
sexual reproduction	5.08	+	8.58E-03
multi-organism reproductive process	5.08	+	8.58E-03
sporulation	13.72	+	9.64E-03
phospholipid catabolic process	17.15	+	7.82E-04
phosphate-containing compound metabolic process	1.25	+	2.09E-02
phosphorus metabolic process	1.26	+	1.68E-02
organophosphate catabolic process	5.41	+	1.88E-02
cellular response to nitrate	17.15	+	6.29E-03
cellular response to reactive nitrogen species	17.15	+	6.29E-03
response to nitrate	11.43	+	1.36E-02
cellular response to inorganic substance	5.72	+	4.86E-02
microsporogenesis	13.72	+	9.64E-03
pollen development	5.49	+	6.60E-03
NADP biosynthetic process	13.72	+	9.64E-03
nicotinamide nucleotide biosynthetic process	5.72	+	4.86E-02
pyridine nucleotide biosynthetic process	5.72	+	4.86E-02
pyridine nucleotide metabolic process	2.18	+	4.49E-02
oxidoreduction coenzyme metabolic process	2.49	+	1.19E-02
coenzyme metabolic process	2.18	+	6.24E-03
coenzyme biosynthetic process	2.24	+	3.97E-02
single-organism biosynthetic process	1.46	+	4.80E-03
nicotinamide nucleotide metabolic process	2.18	+	4.49E-02
embryo development ending in seed dormancy	12.86	+	1.77E-03
embryo development	12.86	+	1.77E-03
seed development	4.29	+	3.41E-02
fruit development	3.96	+	4.16E-02
attachment of GPI anchor to protein	11.43	+	1.36E-02
GPI anchor biosynthetic process	4.68	+	2.74E-02
GPI anchor metabolic process	4.68	+	2.74E-02
lipid biosynthetic process	1.64	+	3.14E-02
phosphatidylinositol biosynthetic process	4.42	+	1.36E-02
apoptotic process	11.43	+	1.36E-02
meiotic DNA double-strand break formation	11.43	+	1.36E-02
protein deneddylation	10.29	+	3.32E-03
histone H3-K9 demethylation	9.80	+	1.82E-02
histone lysine demethylation	7.62	+	2.90E-02

**Gene Ontology biological process**	**Fold Enrichment**	**+/-**	**P value**
histone demethylation	6.86	+	3.51E-02
protein demethylation	6.23	+	4.16E-02
protein dealkylation	6.23	+	4.16E-02
threonine biosynthetic process	9.80	+	1.82E-02
carboxylic acid metabolic process	1.46	+	2.71E-02
oxoacid metabolic process	1.51	+	1.47E-02
threonine metabolic process	8.57	+	2.33E-02
nitrate transport	9.80	+	1.82E-02
7-methylguanosine RNA capping	9.80	+	1.82E-02
anther wall tapetum development	9.80	+	1.82E-02
anther development	6.86	+	3.51E-02
RNA (guanine-N7)-methylation	7.62	+	2.90E-02
regulation of cell proliferation	6.86	+	3.51E-02
DNA catabolic process	6.23	+	4.16E-02
DNA metabolic process	1.63	+	3.28E-02
hexose catabolic process	5.72	+	4.86E-02
single-organism carbohydrate metabolic process	1.80	+	8.19E-03
carbohydrate metabolic process	1.41	+	2.54E-02
single-organism carbohydrate catabolic process	2.33	+	3.35E-02
carbohydrate catabolic process	1.83	+	2.90E-02
3'-UTR-mediated mRNA destabilization	5.72	+	4.86E-02
mRNA destabilization	5.72	+	4.86E-02
regulation of mRNA stability	5.72	+	4.86E-02
RNA destabilization	5.72	+	4.86E-02
glucuronoxylan biosynthetic process	5.72	+	4.86E-02
glucuronoxylan metabolic process	5.72	+	4.86E-02
xylan metabolic process	3.71	+	2.42E-02
hemicellulose metabolic process	3.23	+	4.00E-03
cell wall polysaccharide metabolic process	3.23	+	4.00E-03
polysaccharide metabolic process	2.17	+	1.03E-03
cell wall macromolecule metabolic process	2.45	+	1.86E-02
xylan biosynthetic process	4.90	+	2.43E-02
cell wall polysaccharide biosynthetic process	4.90	+	2.43E-02
cellular polysaccharide biosynthetic process	2.64	+	8.38E-03
cellular polysaccharide metabolic process	2.40	+	2.77E-03
cellular carbohydrate metabolic process	1.87	+	1.19E-02
polysaccharide biosynthetic process	2.35	+	1.20E-02
carbohydrate biosynthetic process	1.75	+	4.65E-02
cellular carbohydrate biosynthetic process	2.07	+	2.62E-02
cell wall macromolecule biosynthetic process	4.47	+	3.06E-02
cellular component macromolecule biosynthetic process	4.47	+	3.06E-02
cell wall biogenesis	2.94	+	4.28E-03
glycogen metabolic process	5.14	+	2.15E-02
cellular glucan metabolic process	2.26	+	1.13E-02
glucan metabolic process	2.38	+	5.73E-03
energy reserve metabolic process	5.14	+	2.15E-02
generation of precursor metabolites and energy	1.67	+	4.69E-02
oxidation-reduction process	1.36	+	6.80E-03
one-carbon metabolic process	5.08	+	8.58E-03
root development	4.47	+	3.06E-02
root system development	4.47	+	3.06E-02
methionine metabolic process	4.29	+	3.41E-02
branched-chain amino acid biosynthetic process	4.12	+	3.77E-02
DNA duplex unwinding	3.57	+	1.42E-02
DNA geometric change	3.43	+	1.66E-02
ribonucleoprotein complex export from nucleus	3.43	+	3.09E-02
protein export from nucleus	3.05	+	4.42E-02
ribonucleoprotein complex localization	3.43	+	3.09E-02
double-strand break repair via homologous recombination	3.27	+	3.59E-02
recombinational repair	3.27	+	3.59E-02
DNA repair	1.95	+	1.52E-02
cellular response to DNA damage stimulus	1.86	+	2.17E-02
response to stress	1.43	+	7.36E-03
DNA recombination	3.99	+	2.79E-04
double-strand break repair	2.72	+	3.90E-02
plant-type cell wall biogenesis	2.72	+	3.90E-02

**Gene Ontology biological process**	**Fold Enrichment**	**+/-**	**P value**
plant-type cell wall organization or biogenesis	1.97	+	3.43E-02
pyruvate metabolic process	2.53	+	2.31E-02
cell division	2.43	+	1.95E-02
microtubule-based process	2.18	+	4.49E-02
hydrogen peroxide catabolic process	2.05	+	4.56E-02
hydrogen peroxide metabolic process	2.05	+	4.56E-02
protein dephosphorylation	1.98	+	3.32E-02
dephosphorylation	1.64	+	4.06E-02
cell wall organization	1.64	+	3.08E-02
external encapsulating structure organization	1.62	+	3.50E-02
regulation of transcription from RNA polymerase II promoter	0.38	-	1.96E-02
regulation of transcription, DNA-templated	0.76	-	3.56E-02
regulation of nucleic acid-templated transcription	0.75	-	3.10E-02
regulation of RNA biosynthetic process	0.75	-	3.10E-02
regulation of RNA metabolic process	0.75	-	2.77E-02
