# Supplementary material for: Molecular evolution and diversification of the GRF transcription factor family
Source: Genet Mol Biol. 2020 Jul 24;43(3):20200080. doi: 10.1590/1678-4685-GMB-2020-0080 (PMC7380329; doi:10.1590/1678-4685-GMB-2020-0080)
Supplement: Supplementary file 8 [file 1415-4757-GMB-43-3-e20200080-suppl6.pdf]

Genetics and Molecular Biology
https://doi.org/10.1590/1678-4685-GMB-2020-0080

Supplementary Material to "Molecular evolution and diversification of the GRF transcription factor family"

Table S6 – Annotation of 1270 putative targets of GRFs in rice genome

OS03G0724600	OS02G0612900	OS02G0674400	OS06G0346600	OS08G0202300	OS10G0499200
OS03G0803700	OS01G0117900	OS11G0260200	OS08G0360100	OS08G0561250	OS11G0173366
OS12G0637100	OS02G0223200	OS01G0348700	OS11G0252400	OS02G0514500	OS10G0190600
OS08G0120500	OS05G0154800	OS06G0681200	OS12G0169400	OS07G0490800	OS05G0358400
OS02G0158500	OS09G0530300	OS10G0537450	OS09G0472700	OS12G0133700	OS09G0418800
OS05G0404000	OS01G0209000	OS07G0125500	OS02G0805300	OS05G0131000	OS04G0331650
OS06G0691800	OS04G0502800	OS12G0443000	OS04G0551500	OS04G0599300	OS11G0644000
OS01G0609501	OS01G0503400	OS07G0502550	OS10G0342650	OS07G0272300	OS07G0588900
OS10G0539200	OS02G0280400	OS05G0207900	OS06G0102750	OS05G0454400	OS07G0564533
OS07G0148800	OS01G0750666	OS01G0919200	OS11G0275900	OS10G0170200	OS08G0190500
OS04G0121200	OS12G0110150	OS03G0312500	OS03G0269700	OS12G0539751	OS02G0465000
OS02G0146400	OS05G0499300	OS10G0480900	OS03G0701900	OS12G0589400	OS04G0530801
OS03G0383800	OS12G0236800	OS05G0481400	OS09G0410300	OS07G0673801	OS03G0803800
OS09G0399502	OS11G0119311	OS02G0184450	OS09G0536300	OS09G0440600	OS12G0284800
OS03G0818400	OS07G0523150	OS02G0299600	OS09G0443400	OS07G0294700	OS10G0148400
OS03G0821900	OS11G0268300	OS05G0393200	OS09G0423500	OS02G0102900	OS07G0599201
OS09G0539500	OS03G0106300	OS09G0439600	OS07G0294600	OS03G0751400	OS05G0534400
OS08G0248300	OS04G0559400	OS09G0407950	OS02G0658300	OS07G0191600	OS03G0193901
OS01G0263300	OS06G0217600	OS02G0621700	OS03G0837100	OS01G0575400	OS10G0345701
OS02G0764700	OS12G0631100	OS04G0450500	OS01G0182900	OS07G0563700	OS02G0756200
OS04G0656800	OS06G0508950	OS01G0897200	OS02G0787250	OS03G0126450	OS03G0643611
OS01G0653800	OS12G0132900	OS02G0128000	OS02G0655750	OS09G0498000	OS02G0806350
OS11G0130800	OS01G0128100	OS07G0567801	OS06G0186400	OS09G0397800	OS04G0623600
OS10G0581000	OS02G0211200	OS03G0805700	OS12G0578600	OS06G0153000	OS04G0679400
OS06G0679750	OS09G0283600	OS03G0742400	OS02G0316200	OS08G0529100	OS08G0307300
OS10G0518400	OS09G0322100	OS05G0390100	OS08G0499300	OS06G0136900	OS10G0359500
OS07G0627000	OS01G0185000	OS05G0198100	OS04G0407350	OS01G0167750	OS07G0591400
OS10G0493600	OS07G0139700	OS01G0223500	OS08G0206700	OS10G0464300	OS04G0423200
OS09G0411500	OS01G0286500	OS03G0770900	OS08G0524600	OS02G0684900	OS01G0714225
OS11G0105750	OS03G0797000	OS01G0531200	OS07G0423700	OS09G0135100	OS05G0326600
OS02G0724600	OS06G0676400	OS01G0238850	OS04G0630551	OS12G0166100	OS02G0574600
OS01G0621900	OS08G0461100	OS09G0432231	OS12G0143300	OS02G0682700	OS03G0846400
OS06G0493700	OS09G0324000	OS10G0146901	OS01G0694000	OS01G0511100	OS01G0824500
OS03G0235400	OS11G0676200	OS05G0137400	OS08G0164300	OS03G0579900	OS01G0207300
OS04G0252850	OS02G0811101	OS01G0118700	OS04G0444050	OS05G0567600	OS03G0213700
OS10G0434650	OS11G0435500	OS10G0159600	OS12G0438300	OS06G0657750	OS03G0130400
OS01G0656400	OS09G0496250	OS06G0298400	OS06G0688300	OS10G0560000	OS05G0150000

OS06G0597200	OS12G0237900	OS11G0133300	OS02G0141100	OS12G0264500	OS10G0559200
OS03G0370800	OS02G0208400	OS10G0130500	OS06G0153100	OS05G0558400	OS07G0644400
OS06G0621100	OS06G0643800	OS03G0604700	OS02G0760000	OS05G0203912	OS03G0243750
OS03G0302800	OS01G0928000	OS11G0591700	OS07G0578333	OS08G0509400	OS11G0578100
OS12G0290600	OS04G0310900	OS05G0443500	OS12G0582666	OS08G0468300	OS07G0597500
OS03G0158000	OS10G0406100	OS03G0745700	OS06G0677600	OS12G0183450	OS07G0667100
OS07G0160050	OS04G0604300	OS03G0105700	OS01G0242500	OS04G0657600	OS01G0164300
OS03G0756000	OS06G0140200	OS02G0604600	OS10G0498800	OS10G0422401	OS05G0564700
OS01G0833500	OS01G0220801	OS02G0467700	OS03G0298300	OS06G0663600	OS07G0187300
OS12G0233300	OS01G0219200	OS01G0279200	OS02G0614966	OS08G0506000	OS11G0188100
OS06G0134750	OS08G0510900	OS08G0379000	OS05G0151300	OS02G0125600	OS07G0623000
OS08G0386900	OS05G0192500	OS03G0221600	OS05G0451200	OS07G0619100	OS12G0503200
OS02G0782500	OS01G0656250	OS04G0581600	OS07G0546400	OS08G0437050	OS02G0106100
OS06G0152400	OS04G0652450	OS10G0341700	OS10G0513300	OS02G0111800	OS07G0255900
OS06G0651300	OS05G0494200	OS10G0519500	OS05G0126501	OS03G0219300	OS05G0507300
OS12G0631600	OS02G0187300	OS07G0577500	OS02G0144800	OS01G0728200	OS02G0115900
OS02G0149500	OS01G0915000	OS10G0570200	OS07G0176200	OS11G0107500	OS05G0110100
OS12G0477200	OS10G0522200	OS02G0780600	OS06G0266266	OS03G0836200	OS08G0285301
OS03G0639233	OS01G0337180	OS06G0627001	OS12G0105300	OS02G0245800	OS01G0159800
OS03G0654500	OS07G0456700	OS03G0200400	OS10G0500600	OS07G0577801	OS12G0580500
OS06G0121200	OS11G0506150	OS06G0350700	OS05G0227901	OS02G0248200	OS08G0374100
OS12G0577900	OS02G0778300	OS11G0183800	OS02G0565500	OS02G0595800	OS03G0134550
OS10G0548200	OS12G0164800	OS03G0283500	OS01G0605400	OS08G0240566	OS08G0149566
OS11G0629600	OS03G0439650	OS01G0844800	OS01G0812050	OS12G0180400	OS05G0204900
OS06G0165600	OS02G0823800	OS10G0406900	OS04G0142850	OS06G0329200	OS02G0184200
OS12G0124700	OS01G0919100	OS10G0524700	OS02G0759500	OS04G0490600	OS07G0678700
OS01G0719100	OS04G0482100	OS08G0187800	OS01G0111000	OS03G0241900	OS12G0529900
OS08G0159700	OS08G0440850	OS11G0514800	OS04G0690932	OS02G0160400	OS06G0688800
OS06G0349800	OS08G0564000	OS11G0676100	OS03G0276800	OS02G0644466	OS09G0305900
OS08G0488100	OS12G0244150	OS03G0234900	OS07G0202000	OS01G0252400	OS07G0641200
OS07G0516600	OS01G0811100	OS04G0644700	OS10G0575600	OS04G0671900	OS03G0609200
OS04G0420801	OS03G0760700	OS01G0861900	OS04G0650600	OS06G0139200	OS01G0167100
OS04G0584750	OS09G0298266	OS08G0564500	OS10G0130200	OS02G0328300	OS12G0502800
OS08G0403733	OS01G0548750	OS02G0704000	OS10G0330600	OS12G0242500	OS06G0161800
OS07G0659100	OS07G0185100	OS06G0233200	OS04G0167800	OS01G0110600	OS12G0411700
OS04G0382300	OS01G0548600	OS05G0137200	OS03G0320850	OS03G0399000	OS03G0247750
OS11G0674400	OS02G0521600	OS04G0640300	OS08G0396200	OS05G0101600	OS03G0391700
OS08G0504600	OS04G0377932	OS04G0321700	OS10G0404500	OS05G0529900	OS09G0401100
OS11G0184100	OS01G0548900	OS04G0629700	OS04G0488400	OS10G0102400	OS01G0276700
OS07G0509300	OS12G0630600	OS05G0150600	OS01G0945666	OS11G0194200	OS03G0255500
OS12G0222650	OS06G0326400	OS02G0327700	OS10G0437700	OS01G0976950	OS09G0386200
OS04G0348100	OS01G0107750	OS03G0228200	OS03G0840500	OS02G0730775	OS08G0139000
OS11G0192400	OS05G0482650	OS06G0212100	OS06G0611000	OS03G0638800	OS03G0111100
OS07G0244200	OS03G0725100	OS05G0276100	OS07G0194500	OS07G0519725	OS04G0648700

OS04G0472200	OS02G0581000	OS01G0772400	OS03G0314700	OS02G0152800	OS04G0488800
OS07G0672175	OS09G0438700	OS10G0557750	OS01G0752100	OS05G0455500	OS12G0124100
OS02G0107900	OS03G0195500	OS03G0737300	OS12G0559200	OS05G0182100	OS12G0628100
OS09G0461500	OS11G0189900	OS05G0464350	OS05G0366900	OS11G0282600	OS07G0123601
OS10G0512500	OS01G0183400	OS04G0313500	OS06G0621800	OS07G0270900	OS01G0872600
OS08G0471300	OS11G0638900	OS03G0208750	OS08G0516500	OS09G0559500	OS09G0482740
OS04G0498800	OS01G0727840	OS01G0680700	OS11G0629566	OS08G0417000	OS11G0144900
OS03G0432100	OS10G0389500	OS04G0308100	OS01G0737100	OS04G0297933	OS10G0578900
OS01G0933075	OS07G0181000	OS03G0638000	OS12G0609500	OS11G0528300	OS06G0712300
OS06G0695350	OS01G0579900	OS07G0599600	OS02G0114200	OS02G0142250	OS08G0351300
OS06G0125400	OS05G0501800	OS02G0741000	OS08G0234200	OS07G0546501	OS10G0421500
OS12G0621300	OS12G0289600	OS01G0566050	OS08G0482100	OS01G0888900	OS02G0176400
OS10G0556100	OS06G0162100	OS08G0100600	OS07G0657300	OS12G0605850	OS01G0905400
OS08G0163900	OS09G0243000	OS04G0675300	OS11G0150801	OS02G0248300	OS01G0514450
OS05G0428000	OS02G0745700	OS01G0635800	OS05G0393100	OS08G0512600	OS07G0178700
OS01G0763850	OS01G0866500	OS04G0526600	OS01G0229400	OS03G0155300	OS07G0237900
OS01G0955000	OS12G0630500	OS01G0585600	OS11G0490300	OS08G0102050	OS04G0204100
OS07G0686950	OS05G0120800	OS04G0320700	OS01G0332700	OS03G0257000	OS04G0524800
OS07G0190000	OS01G0895500	OS05G0591550	OS04G0541900	OS11G0202500	OS01G0215500
OS03G0797300	OS08G0414200	OS06G0179400	OS01G0834100	OS01G0338000	OS12G0161700
OS02G0220000	OS07G0203925	OS09G0311600	OS11G0121000	OS02G0194400	OS07G0665000
OS07G0516650	OS04G0475100	OS07G0605800	OS12G0160650	OS03G0120100	OS07G0184200
OS02G0143400	OS01G0802000	OS03G0181675	OS01G0714100	OS12G0168150	OS04G0373400
OS03G0566700	OS03G0320800	OS04G0306300	OS12G0225150	OS03G0734100	OS05G0395116
OS11G0137050	OS02G0761600	OS07G0105000	OS05G0555450	OS01G0172900	OS08G0424100
OS03G0700700	OS11G0158500	OS06G0186300	OS06G0146700	OS03G0171900	OS08G0105400
OS09G0507701	OS02G0784400	OS07G0435900	OS02G0173100	OS08G0352100	OS04G0174100
OS04G0436400	OS10G0578050	OS12G0129650	OS11G0572500	OS02G0234500	OS03G0104000
OS02G0581900	OS07G0141700	OS03G0311300	OS05G0524400	OS06G0148900	OS01G0564300
OS04G0167875	OS11G0199600	OS04G0580700	OS06G0520733	OS06G0656566	OS04G0401200
OS10G0561000	OS04G0429300	OS05G0358000	OS07G0649300	OS01G0510901	OS10G0150300
OS10G0553900	OS02G0197400	OS03G0180300	OS01G0266500	OS02G0779300	OS11G0474566
OS01G0144000	OS03G0659700	OS09G0481700	OS02G0281150	OS08G0556400	OS01G0670500
OS12G0183100	OS09G0569780	OS02G0776800	OS04G0523812	OS09G0121000	OS10G0142700
OS02G0623600	OS11G0423800	OS01G0654450	OS09G0317700	OS01G0318700	OS02G0709400
OS02G0150450	OS01G0834250	OS08G0561600	OS07G0628700	OS07G0242700	OS01G0706400
OS05G0128350	OS01G0165800	OS11G0170100	OS04G0211900	OS12G0168900	OS07G0137600
OS05G0556650	OS02G0614400	OS04G0622800	OS12G0113001	OS05G0429802	OS01G0771900
OS06G0339800	OS04G0483600	OS02G0106201	OS01G0597701	OS11G0669100	OS04G0590950
OS05G0549700	OS01G0653350	OS08G0119000	OS10G0542750	OS03G0141800	OS04G0623066
OS04G0677650	OS02G0203700	OS08G0550400	OS09G0558100	OS11G0481500	OS11G0707900
OS06G0140800	OS12G0591500	OS05G0508900	OS01G0622033	OS06G0331900	OS02G0153300
OS04G0568800	OS10G0578950	OS09G0481200	OS06G0226500	OS10G0130900	OS02G0793300
OS02G0109400	OS11G0170000	OS05G0267732	OS10G0502400	OS04G0221300	OS04G0578400

OS02G0541300	OS08G0248000	OS04G0433600	OS07G0187000	OS06G0496800	OS06G0207783
OS10G0205300	OS12G0114100	OS10G0363100	OS10G0209700	OS03G0202500	OS01G0115950
OS03G0236800	OS07G0174700	OS07G0208533	OS01G0937600	OS03G0206100	OS03G0328100
OS01G0949400	OS01G0966801	OS01G0899800	OS01G0589100	OS07G0117600	OS08G0206800
OS11G0523500	OS06G0602900	OS08G0544600	OS02G0298200	OS03G0167700	OS07G0675000
OS04G0295200	OS04G0455600	OS04G0508800	OS06G0232300	OS06G0490400	OS03G0815200
OS02G0743500	OS11G0518300	OS11G0300600	OS06G0261300	OS04G0457000	OS01G0591300
OS07G0181500	OS07G0486100	OS01G0145200	OS06G0233000	OS12G0620100	OS10G0363300
OS05G0144200	OS06G0122800	OS08G0335500	OS03G0728800	OS09G0569700	OS01G0786000
OS06G0634700	OS01G0917500	OS01G0513400	OS09G0129400	OS02G0666000	OS06G0531751
OS01G0283700	OS06G0691600	OS04G0175000	OS04G0218900	OS04G0107600	OS05G0238200
OS04G0194000	OS02G0650500	OS02G0771500	OS07G0468100	OS02G0714000	OS12G0580600
OS01G0380800	OS09G0572150	OS03G0800000	OS08G0119900	OS08G0509300	OS04G0450300
OS01G0517900	OS07G0581550	OS10G0102900	OS02G0471500	OS03G0591700	OS01G0870201
OS01G0881966	OS04G0499300	OS10G0565100	OS11G0149300	OS08G0240500	OS10G0570700
OS04G0498000	OS10G0573100	OS02G0202300	OS09G0373100	OS09G0430600	OS01G0253600
OS03G0344900	OS10G0531300	OS03G0231400	OS08G0443600	OS11G0644100	OS01G0851100
OS02G0816900	OS02G0787600	OS02G0708600	OS10G0515400	OS04G0504700	OS01G0799500
OS05G0164900	OS06G0474600	OS04G0528100	OS03G0672300	OS08G0541400	OS05G0411150
OS09G0338500	OS08G0431700	OS04G0639300	OS08G0119300	OS05G0125600	OS03G0167500
OS03G0705433	OS06G0267600	OS10G0471300	OS03G0180000	OS08G0480100	OS11G0616700
OS09G0488750	OS06G0665800	OS01G0276900	OS02G0100250	OS11G0608000	OS08G0207401
OS02G0738350	OS03G0850400	OS07G0506000	OS11G0658900	OS12G0168000	OS04G0682600
OS01G0919950	OS09G0371300	OS08G0154700	OS07G0559100	OS03G0111700	OS01G0919150
OS02G0609500	OS02G0135250	OS10G0422566	OS06G0204700	OS05G0452151	OS03G0795300
OS01G0780500	OS04G0198733	OS06G0653800	OS08G0104400	OS03G0148300	OS09G0487500
OS02G0792600	OS12G0107600	OS09G0453700	OS01G0188400	OS09G0464350	OS10G0401000
OS03G0697466	OS10G0550775	OS01G0151700	OS06G0671800	OS04G0224200	OS07G0545500
OS02G0200800	OS04G0463300	OS06G0701300	OS09G0428750	OS07G0692600	OS03G0784600
OS03G0397500	OS10G0390900	OS01G0135600	OS01G0615300	OS06G0714000	OS01G0837350
OS03G0112600	OS02G0596100	OS06G0328200	OS05G0390500	OS08G0471000	OS07G0113000
OS03G0421800	OS01G0682200	OS08G0341700	OS03G0418800	OS01G0183300	OS03G0857200
OS01G0301300	OS02G0630000	OS09G0455300	OS03G0839900	OS11G0441900	OS09G0529300
OS05G0394301	OS06G0199800	OS06G0514700	OS12G0453500	OS08G0386125	OS05G0554500
OS02G0562300	OS08G0560000	OS09G0492700	OS02G0564400	OS03G0121300	OS12G0137600
OS03G0213400	OS10G0474900	OS06G0705100	OS12G0458900	OS12G0443800	OS12G0498300
OS01G0763600	OS02G0112600	OS12G0238400	OS04G0675500	OS12G0133850	OS05G0194000
OS02G0685350	OS01G0917100	OS11G0546300	OS10G0412800	OS06G0665500	OS04G0468600
OS08G0208700	OS06G0695001	OS01G0295900	OS10G0561900	OS02G0762800	OS09G0498800
OS07G0248800	OS01G0267600	OS08G0280200	OS10G0484400	OS02G0614800	OS02G0188600
OS06G0314100	OS06G0708400	OS07G0173400	OS01G0173000	OS05G0586200	OS08G0352400
OS06G0242000	OS10G0155700	OS04G0189800	OS05G0440250	OS07G0513600	OS10G0503600
OS05G0524100	OS04G0583800	OS01G0210200	OS04G0479000	OS12G0614600	OS11G0163800
OS12G0148400	OS03G0713400	OS02G0832150	OS07G0475400	OS03G0268250	OS05G0149000

OS06G0652000	OS03G0245500	OS04G0504500	OS10G0539900	OS07G0681500	OS04G0177500
OS06G0682850	OS05G0100008	OS03G0325500	OS08G0226400	OS02G0463401	OS10G0547000
OS02G0788800	OS01G0273200	OS03G0301500	OS03G0755000	OS01G0663800	OS01G0534800
OS11G0682600	OS05G0153132	OS04G0404400	OS03G0412850	OS05G0586500	OS01G0619000
OS05G0143500	OS05G0396700	OS03G0286800	OS05G0509500	OS01G0693700	OS01G0808100
OS01G0703300	OS04G0663200	OS01G0229000	OS07G0113600	OS11G0168500	OS01G0609300
OS08G0446700	OS11G0245650	OS12G0256900	OS11G0346016	OS08G0195900	OS10G0346600
OS04G0620000	OS09G0252000	OS10G0100500	OS12G0622500	OS10G0417800	OS01G0920200
OS09G0442800	OS11G0184900	OS02G0755000	OS08G0194501	OS04G0350300	OS12G0545400
OS08G0530033	OS02G0217600	OS07G0204000	OS07G0666750	OS10G0462300	OS12G0172400
OS01G0570601	OS06G0609800	OS08G0558400	OS11G0578066	OS02G0607100	OS01G0915400
OS03G0593200	OS03G0422600	OS10G0517050	OS06G0118301	OS08G0108925	OS10G0381601
OS02G0832301	OS01G0934300	OS08G0250100	OS04G0627400	OS09G0250800	OS04G0154000
OS08G0425100	OS05G0589000	OS02G0299000	OS02G0829100	OS08G0326300	OS08G0353700
OS05G0269100	OS01G0757500	OS07G0124000	OS03G0742900	OS06G0716467	OS02G0117400
OS02G0624350	OS06G0325500	OS03G0757900	OS09G0460000	OS07G0476401	OS11G0522900
OS08G0152300	OS12G0591300	OS02G0569400	OS11G0552100	OS11G0110033	OS03G0152800
OS08G0178300	OS09G0441600	OS02G0112600	OS01G0928200	OS06G0133500	OS05G0593200
OS05G0110600	OS04G0480700	OS04G0633400	OS11G0296001	OS03G0603500	OS11G0654000
OS03G0102366	OS06G0257600	OS10G0135100	OS04G0589200	OS10G0580200	OS02G0658400
OS08G0562700	OS02G0807800	OS05G0141400	OS03G0826600	OS06G0602400	OS01G0631700
OS07G0457500	OS07G0694300	OS02G0593700	OS03G0603100	OS04G0630500	OS11G0707100
OS01G0312600	OS08G0296600	OS10G0189100	OS12G0507700	OS01G0659800	OS10G0517500
OS02G0101800	OS05G0157300	OS11G0226400	OS04G0105200	OS02G0470100	OS07G0578700
OS06G0115600	OS11G0695000	OS08G0465400	OS03G0142800	OS07G0491600	OS07G0200000
OS03G0210100	OS03G0825700	OS05G0564601	OS06G0314600	OS01G0864500	OS06G0287200
OS05G0388400	OS09G0462875	OS04G0688000	OS06G0102100	OS11G0572800	OS01G0134700
OS08G0100800	OS01G0199400	OS01G0390300	OS01G0977600	OS04G0677100	OS01G0371300
OS08G0520900	OS05G0528600	OS07G0574500	OS03G0206750	OS05G0363951	OS01G0555200
OS04G0608500	OS10G0485100	OS08G0153750	OS06G0660400	OS04G0507700	OS01G0823400
OS06G0318400	OS09G0469400	OS04G0209450	OS08G0518100	OS07G0245100	OS12G0153700
OS06G0237800	OS02G0595200	OS10G0560451	OS10G0420332	OS07G0167700	OS02G0624300
OS03G0135300	OS11G0231600	OS05G0486100	OS04G0463600	OS04G0589900	OS08G0322550
OS01G0793500	OS09G0306632	OS03G0330200	OS06G0495500	OS06G0695900	OS01G0258600
OS07G0496401	OS04G0503550	OS09G0393200	OS07G0657900	OS01G0237200	OS02G0629600
OS01G0736500	OS02G0727500	OS06G0545601	OS05G0213900	OS05G0202200	OS06G0298100
OS10G0460000	OS11G0550900	OS01G0246601	OS03G0165300	OS01G0544450	OS09G0444500
OS05G0460200	OS03G0232600	OS07G0448100	OS02G0527500	OS12G0620400	OS08G0492100
OS03G0315400	OS09G0457532	OS03G0270800	OS11G0233201	OS03G0396900	OS03G0166600
OS07G0114500	OS02G0814200	OS08G0478100	OS09G0545100	OS01G0844000	OS03G0731800
OS07G0300100	OS03G0645200	OS09G0545250	OS04G0410600	OS02G0794300	OS03G0321100
OS03G0816300	OS04G0398300	OS01G0182300	OS01G0735450	OS04G0421800	
OS08G0276400	OS06G0296900	OS08G0559300	OS07G0123533	OS05G0150800	
